# N^6^-Methyladenosine on Key Messenger RNAs Governs Reproductive Development and Metabolic Adaptation in Human Blood Fluke

**DOI:** 10.34133/research.1328

**Published:** 2026-07-01

**Authors:** Bikash Ranjan Giri, Zihao Zhang, Lu Liu, Chuantao Fang, Shiyu Zhu, Mengting Qiu, Omri Wurtzel, Cizhong Jiang, Guofeng Cheng

**Affiliations:** ^1^ Shanghai Tenth People’s Hospital, Institute for Infectious Diseases and Vaccine Development, School of Medicine, School of Life Sciences and Technology, Tongji University, Shanghai, China.; ^2^ Affiliated Shanghai Blue Cross Brain Hospital, Tongji University School of Medicine, Shanghai, China.; ^3^ Clinical Center for Brain and Spinal Cord Research, Tongji University, Shanghai, China.; ^4^ School of Biotechnology Jiangsu University of Science and Technology, Zhen Jiang, China.; ^5^ The School of Neurobiology, Biochemistry & Biophysics, George S. Wise Faculty of Life Sciences, Tel Aviv University, Tel Aviv, Israel.; ^6^ Key Laboratory of Spine and Spinal Cord Injury Repair and Regeneration of the Ministry of Education, Orthopaedic Department of Tongji Hospital, School of Life Sciences and Technology, Tongji University, Shanghai, China.

## Abstract

Schistosomiasis, a neglected tropical disease, affects more than 250 million people worldwide. The pathology and transmission of schistosomiasis rely on the parasite’s extended survival and massive egg output. While N^6^-methyladenosine (m^6^A) messenger RNA modification is an important epigenetic regulator, its functional roles in pathogenic helminths remain entirely unexplored. Here, we comprehensively characterize the m^6^A landscape in the human blood fluke *Schistosoma japonicum*. We generated high-resolution transcriptome-wide m^6^A maps for adult male and female parasites, revealing distinct sex-specific methylation patterns. Functional investigation of the conserved m^6^A writer complex demonstrated that m^6^A methyltransferase-like-3/14 (METTL3/14)-mediated methylation is indispensable for female reproductive development and oviposition. In males, m^6^A modification is essential for maintaining tegumental integrity, metabolic homeostasis, and motility. Mechanistically, integrated methylated RNA immunoprecipitation sequencing and RNA sequencing analyses following METTL3 knockdown identified key m^6^A-modified transcripts, including glutamine synthetase, which underpin the observed phenotypic defects. Our study provides the first functional evidence of m^6^A-mediated epitranscriptomic regulation in a parasitic helminth, establishing it as a master regulator of reproductive and somatic development and revealing the m^6^A pathway as a potentially important target for developing novel strategies against schistosomiasis.

## Introduction

Schistosomiasis, a neglected tropical disease, affects over 250 million people globally and is primarily caused by *Schistosoma mansoni*, *Schistosoma haematobium*, and *Schistosoma japonicum* [1]. Additionally, *S. japonicum* is endemic to Southeast Asia, including China, the Philippines, and parts of Thailand [1]. The complex life cycle of *Schistosoma* involves developmental transitions in freshwater snails and mammalian definitive hosts, where sexually dimorphic adults pair and produce hundreds of eggs daily [2]. Moreover, adult worms can live in definitive hosts up to several decades [3]. The accumulated egg-induced host immune reactions result in the development of pathology, morbidity, and mortality related to schistosomiasis [4]. Deciphering the molecular mechanisms sustaining schistosome parasitism and egg production is thus crucial for identifying effective anthelmintic targets and developing novel interventions against schistosomiasis.

N^6^-Methyladenosine (m^6^A), recognized as the most prevalent messenger RNA (mRNA) modification in eukaryotes, regulates critical RNA processes such as splicing [5], nuclear export [6], translation [7], and decay [8]. For example, in human, over 12,000 m^6^A sites occur in >7,000 genes [9] that globally modulate posttranscriptional gene expression [10] and RNA metabolism [11]. Accumulated evidence indicates that this RNA modification regulates diverse cellular processes across eukaryotes, including unicellular parasites [12] and mammals [10] as well as many diseases [13,14]. In protozoans such as *Plasmodium falciparum*, an m^6^A methyltransferase has been identified as a regulator of mRNA stability/translational efficiency [15] and YTH (YT521-B homology) domain proteins have been found to regulate protein translation through interaction with m^6^A-modified mRNA [16,17]. In *Toxoplasma gondii*, several members of m^6^A epitranscriptome including m^6^A methyltransferase-like-3 (METTL3), Wilms-tumor-1 associated protein (WTAP), and YTH1 have been shown to contribute to parasite viability by regulating mRNA 3′ ends [18]. In *Trypanosoma brucei*, m^6^A modification in poly(A) tails has been shown to stabilize the transcripts of variant surface glycoproteins [12]. In free-living planarian flatworms, m^6^A was demonstrated to be a key regulator of stem cell biology [19,20]. However, whether m^6^A modification exists in pathogenic helminths and how it contributes to their pathogenicity remains unknown. m^6^A modification is catalyzed by a conserved methyltransferase complex [21]. The complex includes METTL3 [22,23], and a scaffold protein, m^6^A methyltransferase-like-14 (METTL14) [22,23]. In addition, an adaptor protein, WTAP, contributes to the complex localization to nuclear speckles for coordinated mRNA processing [24]. Although other proteins can assemble with the m^6^A methyltransferase complex in mammalian cells, accumulated evidence indicates that a conserved m^6^A core complex contains METTL3, METTL14, and WTAP in eukaryotes [8]. In addition, Vir-like m^6^A methyltransferase-associated (VIRMA) was shown to be important for m^6^A modification [19,25]. Nevertheless, whether *Schistosoma* presents a similar factor for m^6^A modification remains under characterization.

In the study, we demonstrate the critical roles of m^6^A modification in *S. japonicum*. We systemically mapped m^6^A modifications across the *S. japonicum* transcriptome and elucidated the key biological processes that are regulated by its m^6^A writers. Through functional analysis, we found that *Mettl3*/*Mettl14* is required in *S. japonicum* females for ovarian and vitellocyte development, as well as oviposition. In males, the m^6^A writer is involved in metabolic reprogramming essential for survival. Our findings reveal that m^6^A is an underappreciated regulator in *S. japonicum*, pointing to new potential targets for schistosomiasis control and providing important insights into a layer of epigenetic regulation during worm reproductive development.

## Results

### Identification of m^6^A modification in *S. japonicum*

**Fig. 1.**
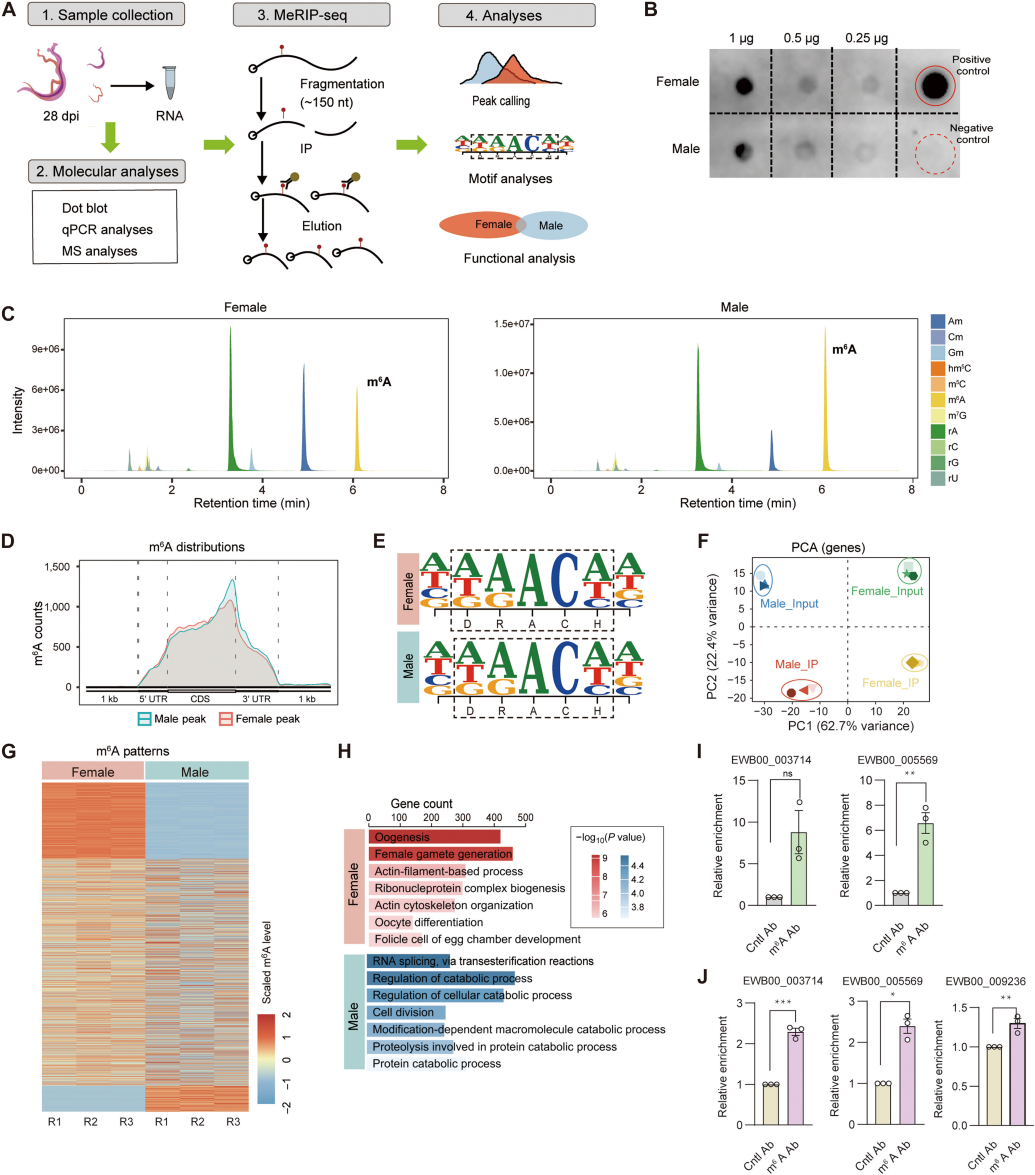
Identification of N^6^-methyladenosine (m^6^A) modification in *Schistosoma japonicum*. (A) Experimental workflow of m^6^A-modified messenger RNA (mRNA) identification in *S. japonicum*. (B) Dot blot analysis of m^6^A modifications in total RNAs isolated from adult males and females of *S. japonicum*. (C) Liquid chromatography coupled with triple quadrupole mass spectrometry (LC–MS/MS) analysis of modified nucleotides in *S. japonicum* mRNA. (D) Distributions of m^6^A peaks across mRNA transcripts in *S. japonicum* females and males. Peaks were mapped relative to the coding sequence (CDS), 5′ untranslated region (5′ UTR), 3′ untranslated region (3′ UTR), and flanking regions (±1 kb). Elevated m^6^A levels were detected near the 3′ UTR in both sexes, with a subtle increase observed in males. (E) Sequence logos representing nucleotide frequency bias flanking m^6^A sites in female and male *S. japonicum* mRNA. Logos depict sequence conservation from positions −3 to +3 nucleotides (nt) relative to the central m^6^A modification site. The size of each nucleotide symbol indicates the observed frequency at that position. The degenerate consensus sequence corresponding to each logo is presented below. (F) Principal component analysis (PCA) of methylated RNA immunoprecipitation sequencing (MeRIP-seq) results. PCA plot of principal components of the MeRIP-seq results with 3 biological replicates for each sex of *S. japonicum*. (G) Heatmap showing distinct m^6^A methylation patterns between females and males. R1, R2, and R3 represent different biological replicates. (H) Gene Ontology (GO) enrichment of biological processes for hypermethylated mRNA in male and female worms. (I) Validation of m^6^A enrichment in female schistosome transcripts via methylated RNA immunoprecipitation followed by real-time quantitative reverse transcription polymerase chain reaction (MeRIP–RT-qPCR). Data illustrate representative results indicating mean ± standard deviation (SD) from an experiment conducted in triplicate. (J) Validation of m^6^A enrichment in male schistosome transcripts via MeRIP–RT-qPCR. Data illustrate representative results indicating mean and SD obtained from an experiment conducted in triplicate. For panels (I) and (J): ns, no significance; *P < 0.05; **P < 0.01; ***P < 0.001.

Prior to this study, the presence of m^6^A in *Schistosoma* remained unexamined. To address this, we employed multiple approaches to detect m^6^A in isolated RNA (Fig. 1A). First, we performed dot blot analysis on serially diluted total RNAs isolated from adult males and females using an anti-m^6^A antibody, confirming the presence of m^6^A (Fig. 1B). Since m^6^A is also known to occur at specific positions on ribosomal RNA [26], we next purified mRNA, hydrolyzed it to mononucleotides, and quantified RNA modifications using liquid chromatography coupled with triple quadrupole mass spectrometry (LC–MS/MS) (Fig. 1C). This high-throughput analysis detected multiple mRNA modifications in both sexes, including riboadenosine, 2′-*O*-methyladenosine, m^6^A, 5-methylcytidine, N7-methylguanosine, 5-hydroxymethylcytidine, and riboguanosine (Fig. 1C). Notably, m^6^A displayed the highest peak intensity and was the most enriched among the internal mRNA modifications detected (Fig. 1C), demonstrating its abundance in *S. japonicum* mRNAs.

To map m^6^A sites transcriptome-wide, we carried out methylated RNA immunoprecipitation sequencing (MeRIP-seq) on adult males and females. We constructed strand-specific RNA sequencing (RNA-seq) libraries from both m^6^A-immunoprecipitated (m^6^A-IP) RNA and input mRNA controls. Integrated analysis showed that m^6^A peaks were preferentially distributed near the 3′ end of transcripts and were predominantly located within coding sequence regions (Fig. 1D), a pattern consistent with those observed in metazoans [27] and plants [28]. Furthermore, sequence motif analysis identified DRACH as a substantially enriched motif within m^6^A peaks. This motif (Fig. 1E) resembles the conserved RGACH (where R = G/A and H = A/C/U) and DRACH (D = G/A/U, R = G/A, and H = C/A/U) motifs found in yeast [29], humans [30], and planarians [31].

We next investigated sex-biased m^6^A modifications by comparing peaks between males and females (Fig. 1F). Using 3 biological replicates per sex and applying a stringent significance threshold (*P* ≤ 1e−10), we identified 43,013 high-confidence m^6^A peaks in males and 70,851 in females (Fig. 1G, Fig. S1, and Tables S2 and S3). Following Gene Ontology (GO) enrichment analysis, we found that m^6^A-modified transcripts revealed distinct functional associations between the sexes. Male-enriched m^6^A peaks were linked to the regulation of catabolism and autophagy-related processes, whereas female-enriched peaks were associated with oogenesis, actin-filament-based processes, and cytoskeleton organization (Fig. 1H). To independently validate the identified m^6^A sites, we performed methylated RNA immunoprecipitation (MeRIP) followed by real-time quantitative reverse transcription polymerase chain reaction (RT-qPCR) on RNA purified from adult males and females, which confirmed the enrichment of selected m^6^A-containing transcripts (Fig. 1I and J). In summary, these results demonstrate that m^6^A is an abundant mRNA modification in *S. japonicum* and exhibits marked sex-specific distribution and functional enrichment, suggesting distinct roles in female reproductive and developmental regulation versus male catabolic processes.

### Characterization of m^6^A writers in *S. japonicum*

**Fig. 2.**
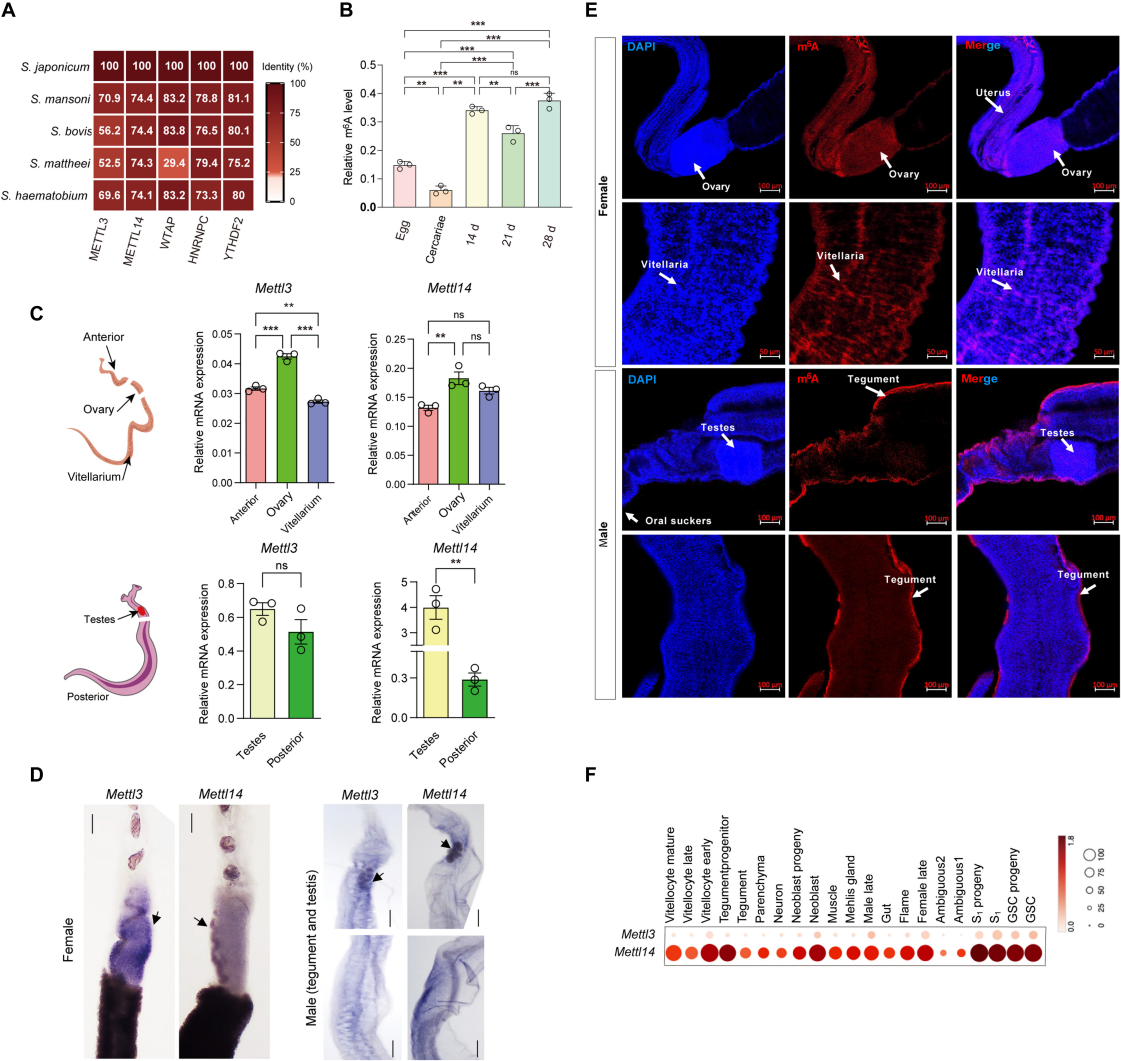
Characterization of *Schistosoma japonicum* N^6^-methyladenosine (m^6^A) writers. (A) m^6^A writers and readers in *S. japonicum* and their identities among different *Schistosoma*. Candidate proteins were analyzed along with their orthologs in other species using the BioMart database. Ortholog relationships were defined by Ensembl standards, with a sequence conservation threshold of ≥25%. (B) Quantification of m^6^A levels for the total RNAs extracted from different developmental stages of worms. Data are presented as the mean ± SD of the representative results of 3 independent experiments. (C) Real-time quantitative reverse transcription polymerase chain reaction (RT-qPCR) analysis of the transcript levels of *Mettl3*/*Mettl14* in dissected worm segments. (Left) Diagrams illustrate the dissection of female (into 3 parts) and male (into 2 parts) worms. (Right) RT-qPCR analysis shows the transcript levels of *Mettl3* and *Mettl14* in the corresponding dissected segments. Data are presented as the mean ± SD of the representative results of 3 independent experiments. For panels (B) and (C): ns, no significance; **P < 0.01; ***P < 0.001. (D) Whole-worm in situ hybridization (WISH) analyses showing the localizations of *Mettl3*/*Mettl14* in the ovary and vitelline duct of female worms; WISH analyses showing the localizations of *Mettl3*/*Mettl14* in the anterior portion of males and its teguments. Data are representative results of at least 20 investigated worms. Arrows indicate the ovary in females and the testis in males. Scale bars = 200 μm. (E) Immunofluorescence analysis of m^6^A-modified RNA in *S. japonicum* females showing high enrichment in the vitelline duct and vitellaria, counterstained with 4′,6-diamidino-2-phenylindole (DAPI; blue); immunofluorescence m^6^A-modified RNA in *S. japonicum* males showing high expression in the tegument, counterstained with DAPI (blue). Data are representative results of 25 to 30 investigated worms. (F) Single-cell RNA sequencing (RNA-seq) data showing the transcript enrichment of m^6^A writers in different cell populations of *S. japonicum*.

METTL3 and METTL14, as core components of the m^6^A methyltransferase complex, are highly evolutionarily conserved among different species [32]. To identify the corresponding orthologs in *S. japonicum*, we performed sequence comparisons with established animal model systems (Fig. 2A and Fig. S2). Given that schistosomes undergo multiple developmental stages, we measured m^6^A levels across different life stages using an m^6^A-specific enzyme-linked immunosorbent assay. This assay revealed a significant increase in m^6^A abundance in adult worms compared to that in earlier stages (Fig. 2B). Firstly, we dissected female *S. japonicum* into 3 anatomical segments. RT-qPCR analysis showed that transcripts of *Mettl3* and *Mettl14* were most abundant in the region containing the ovaries (Fig. 2C). This expression pattern was subsequently confirmed by whole-worm in situ hybridization (WISH), which revealed strong signals for both genes in the ovaries and vitellaria (Fig. 2D). Consistent with these findings, immunofluorescence staining using an anti-m^6^A antibody showed elevated m^6^A signals in these female reproductive tissues (Fig. 2E). In male worms, dissection into 2 segments followed by RT-qPCR indicated higher *Mettl3*/*Mettl14* expression in the gonadal region compared to that in the posterior part (Fig. 2C). WISH analysis detected widespread expression throughout the tegument and body, with particularly strong signals in the testis (Fig. 2D). Accordingly, immunofluorescence also revealed substantial m^6^A accumulation in male tegument (Fig. 2E). We further examined the expressions of *Mettl3* and *Mettl14* using a single-cell RNA-seq dataset from *S. japonicum* and indicated that both genes were predominantly expressed in germline stem cells (GSCs) and their progeny, S_1_/S_1_ progeny, female late-stage cells, male late-stage cells, neoblasts, tegumental progenitors, and vitellocytes (early, late, and mature) (Fig. 2F), corroborating the spatial expression patterns observed with WISH and immunofluorescence. Together, these results demonstrate a sexually dimorphic distribution of METTL3 and METTL14 in *S. japonicum*, with enrichment in female reproductive organs versus male teguments and testes. These findings align well with the sexually biased functional terms identified in the GO analysis of m^6^A-modified transcripts (Fig. 1H), supporting a role for m^6^A in sex-specific biological processes.

### m^6^A writers regulate reproductive development in *S. japonicum* females

**Fig. 3.**
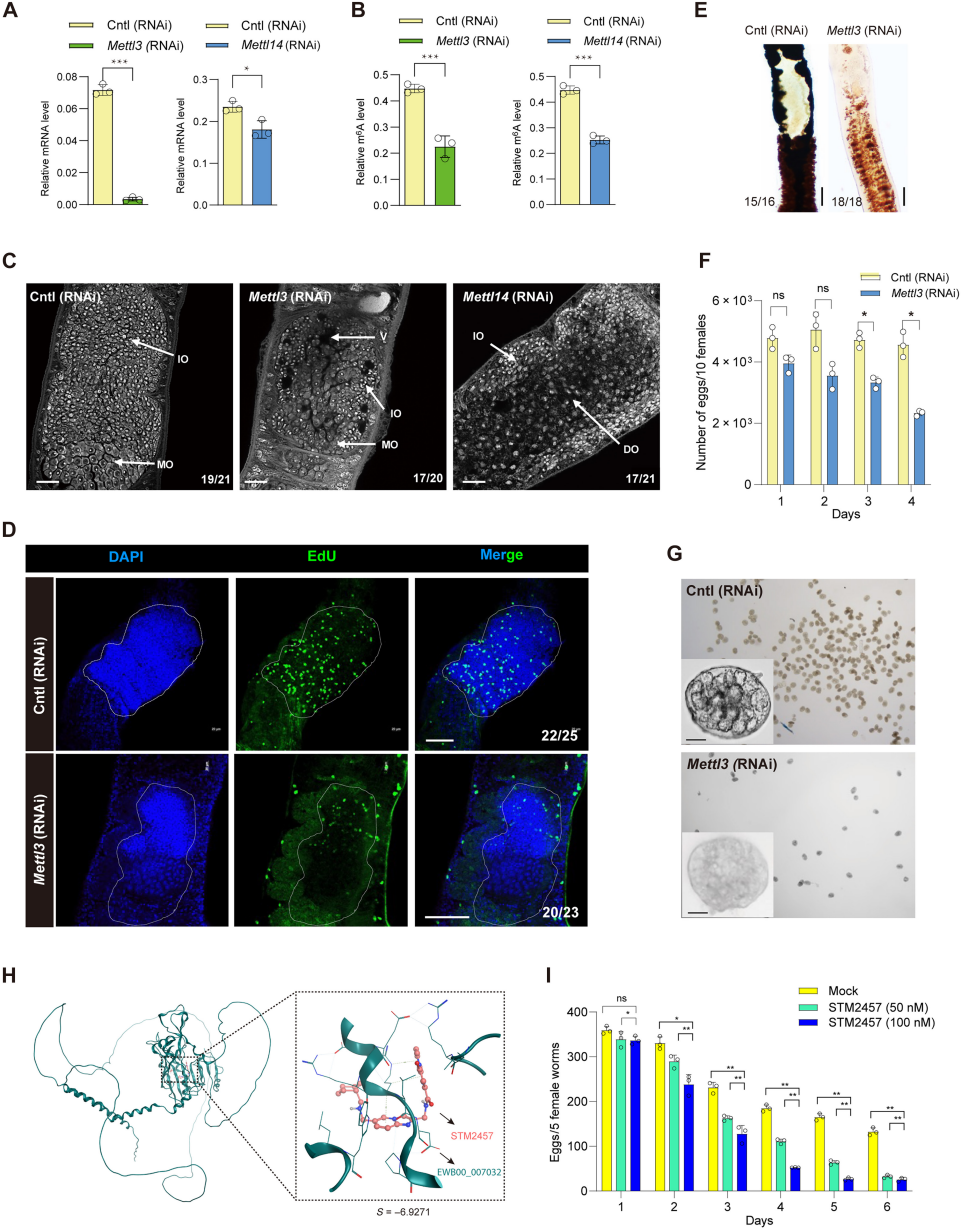
N^6^-Methyladenosine (m^6^A) writers regulate reproductive development in *Schistosoma japonicum* females. (A) Validation of *Mettl3*/*Mettl14* knockdown (KD) efficiency in female schistosomes via real-time quantitative reverse transcription polymerase chain reaction (RT-qPCR). Data illustrate representative results indicating mean and standard deviation obtained from an experiment conducted in triplicate. *P < 0.05; ***P < 0.001. (B) *Mettl3*/*Mettl14* suppression led to reduced m^6^A levels of treated worms. Data illustrate representative results indicating mean and standard deviation obtained from an experiment conducted in triplicate. ***P < 0.001. (C) Suppression of *Mettl3* and *Mettl14* altered the morphology of female ovary. IO, immature oocytes; MO, mature oocytes; V, vacuoles; DO, damaged oocytes. The fraction in the lower right corner of each panel indicates the number of worms exhibiting the shown phenotype/total number of worms examined. Scale bar = 40 μm. (D) *Mettl3* inhibition led to decreased cell proliferation in the ovary of females. The fraction in the lower right corner of each panel indicates the number of worms displaying the representative phenotype shown, out of the total number of worms examined. Scale bar = 50 μm. (E) Fast Blue BB staining analysis of vitellocyte morphology in *Mettl3*-suppressed worms. The fraction in the lower left corner of each panel indicates the number of worms displaying the representative phenotype shown, out of the total number of worms examined. Scale bar = 40 μm. (F) Reduced number of eggs produced in *Mettl3*-suppressed females. Data illustrate representative results as mean ± standard error (SE) derived from the experiments of 3 replicates. An unpaired Student *t* test was used to analyze the significant differences between the control and RNA interference (RNAi)-treated groups (*P < 0.05). (G) Images of produced eggs in *Mettl3*-KD females and control females. Scale bar = 20 μm. (H) Molecular docking analysis of the conformation between sjMETTL3 (EWB00_007032) and the small-molecule inhibitor STM2457. (I) Decreased number of eggs produced in females treated with STM2457. Data illustrate representative results as mean ± SE derived from the experiments of 3 replicates. An unpaired Student *t* test was used to analyze the significant differences between the control and RNAi-treated groups. ns, no significance; *P < 0.05; **P < 0.01.

To directly assess the functional role of m^6^A modification in *S. japonicum*, we designed 3 small interfering RNAs (siRNAs) targeting either *Mettl3* or *Mettl14* and then selected the most effective candidates (*Mettl3*-1412 and *Mettl14*-851) based on knockdown (KD) efficiency in cultured parasites (Fig. S3). Electroporation-mediated delivery of these siRNAs into cultured adult females resulted in a significant reduction in the transcript levels of *Mettl3* and *Mettl14* (Fig. 3A), accompanied by a global decrease in m^6^A abundance (Fig. 3B). Based on the available antibodies, the decreased expression of METTL3 at the protein level was further validated by Western blot (Fig. S4). At day 4 post-KD, we observed marked ovarian developmental defects (Fig. 3C and Fig. S5A to C). Whereas control ovaries displayed mature oocytes (MO) posteriorly and immature oocytes (IO) anteriorly, *Mettl3*- or *Mettl14*-KD worms exhibited widespread oocyte disruption (DO) and vacuolization (V), with more severe defects following *Mettl3* KD (Fig. 3C and Fig. S5B and C). We hypothesized that reduced cell production contributed to the degeneration of the ovary. Indeed, 5-ethynyl-2′-deoxyuridine (EdU) labeling revealed a marked reduction in proliferating cells after *Mettl3* KD (Fig. 3D). Moreover, Fast Blue BB staining revealed impaired vitellocyte development (Fig. 3E). Importantly, these defects were not limited to abnormal anatomy: following *mettl3* and *mettl14* suppression, we quantified egg production and observed significant egg output reduction at 3 to 4 d posttreatment (Fig. 3F and G). To further validate the results, we used STM2457, a METTL3 inhibitor, to treat in vitro cultured females. Upon the confirmation of their substantially binding affinity by molecular docking (Fig. 3H), in vitro culture females were treated with this chemical inhibitor under different concentrations. Then, we examined the number of eggs produced during the time course of the treatment and found a significant reduction in egg production (Fig. 3I). Overall, these results demonstrated that m^6^A modification is an essential regulator of female gonad development, oviposition, and egg production in female *S. japonicum*.

### Inhibition of *S. japonicum Mettl3* in infected animals reduces egg production and host pathology

**Fig. 4.**
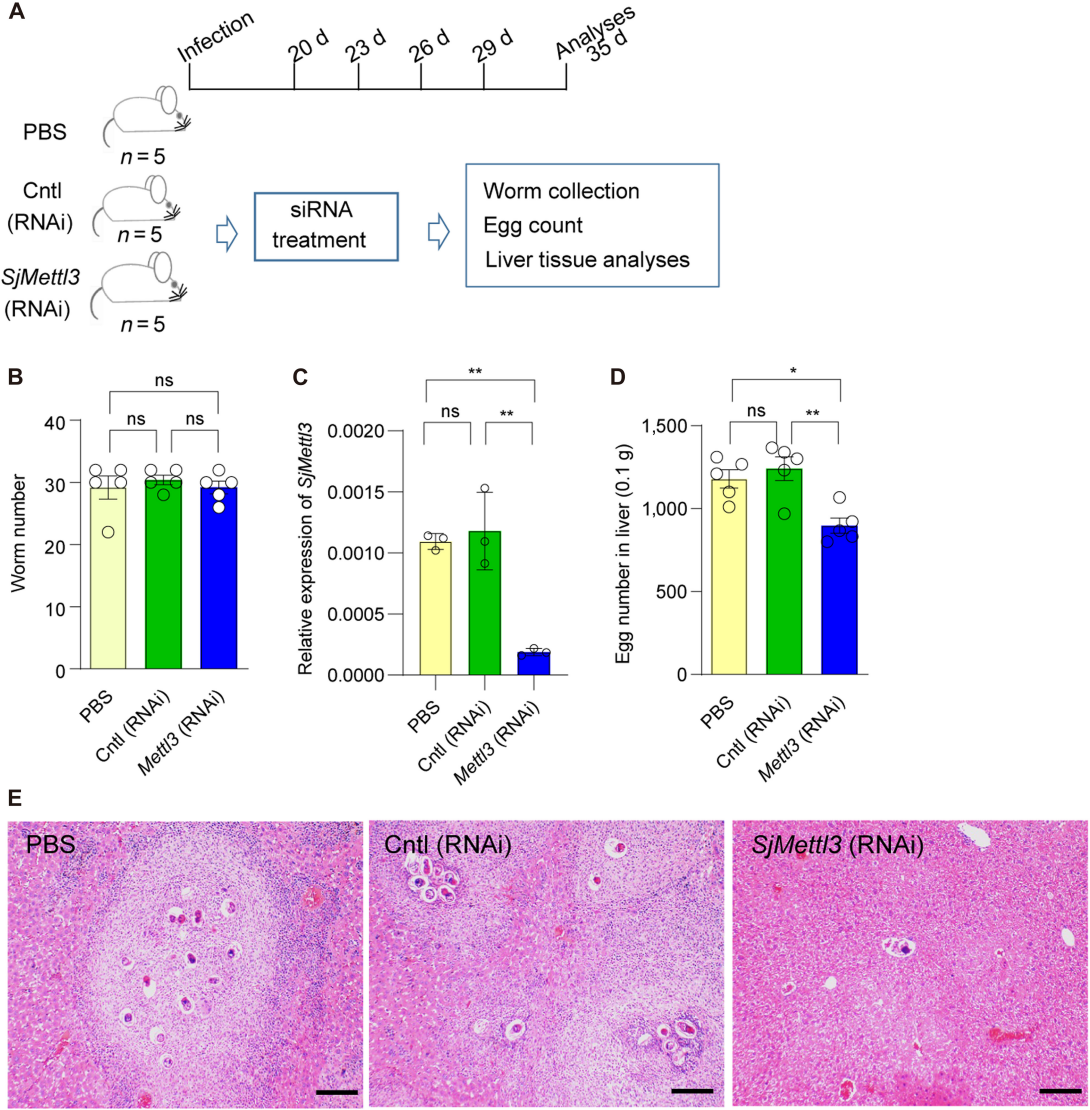
*Schistosoma japonicum Mettl3* (*SjMettl3*) inhibition reduces egg production and host pathology. (A) Schematic showing *SjMettl3* inhibition in mice infected with *S. japonicum* cercariae. Five mice (*n* = 5) for each group. (B) Evaluation of worm burden in infected mice at 35 d postinfection (dpi) upon small interfering RNAs (siRNA) treatment. Data show the mean and standard deviation (*n* = 5). (C) Real-time quantitative reverse transcription polymerase chain reaction (RT-qPCR) analyses of the transcript level of *SjMettl3* in the remaining worms collected from administrated mice. Data illustrate representative results indicating mean and standard deviation from an experiment conducted in triplicate. (D) Effect of siRNA treatment on egg deposition in the liver of infected mice. Data show the mean and standard deviation (*n* = 5) that eggs counted from randomly selected tissues from the same liver lobe (weight: 0.1 g) for each mouse. (E) Histopathological analysis of liver tissues from mice treated with phosphate-buffered saline (PBS), control siRNA, and *SjMettl3* siRNA. Data show representative results from 3 independent analyses. Scale bar = 50 μm. For panels (B) to (D): ns, no significance; *P < 0.05; **P < 0.01.

To validate whether *S. japonicum Mettl3* (*SjMettl3*) suppression in infected animals reduces egg production and host pathology in animal model, we administered *SjMettl3* siRNA into the infected mice (Fig. 4A). Upon 4 injections, at 35 d postinfection (dpi), we perfused worms from the mice and found that there was no statistical significance for reducing worm recovery (Fig. 4B). Next, we used RT-qPCR to determine whether the transcript levels of *SjMettl3* were altered in the collected worms. The result indicated that the transcript level of *SjMettl3* was significantly decreased (Fig. 4C). It is worth noting that the number of eggs deposited in murine livers was significantly decreased (Fig. 4D) and pathological analyses indicated that egg-induced granulomata mitigate in the mice when applying *SjMettl3* siRNA (Fig. 4E).

### Mechanism of the m^6^A writer for regulating ovarian development and oviposition

**Fig. 5.**
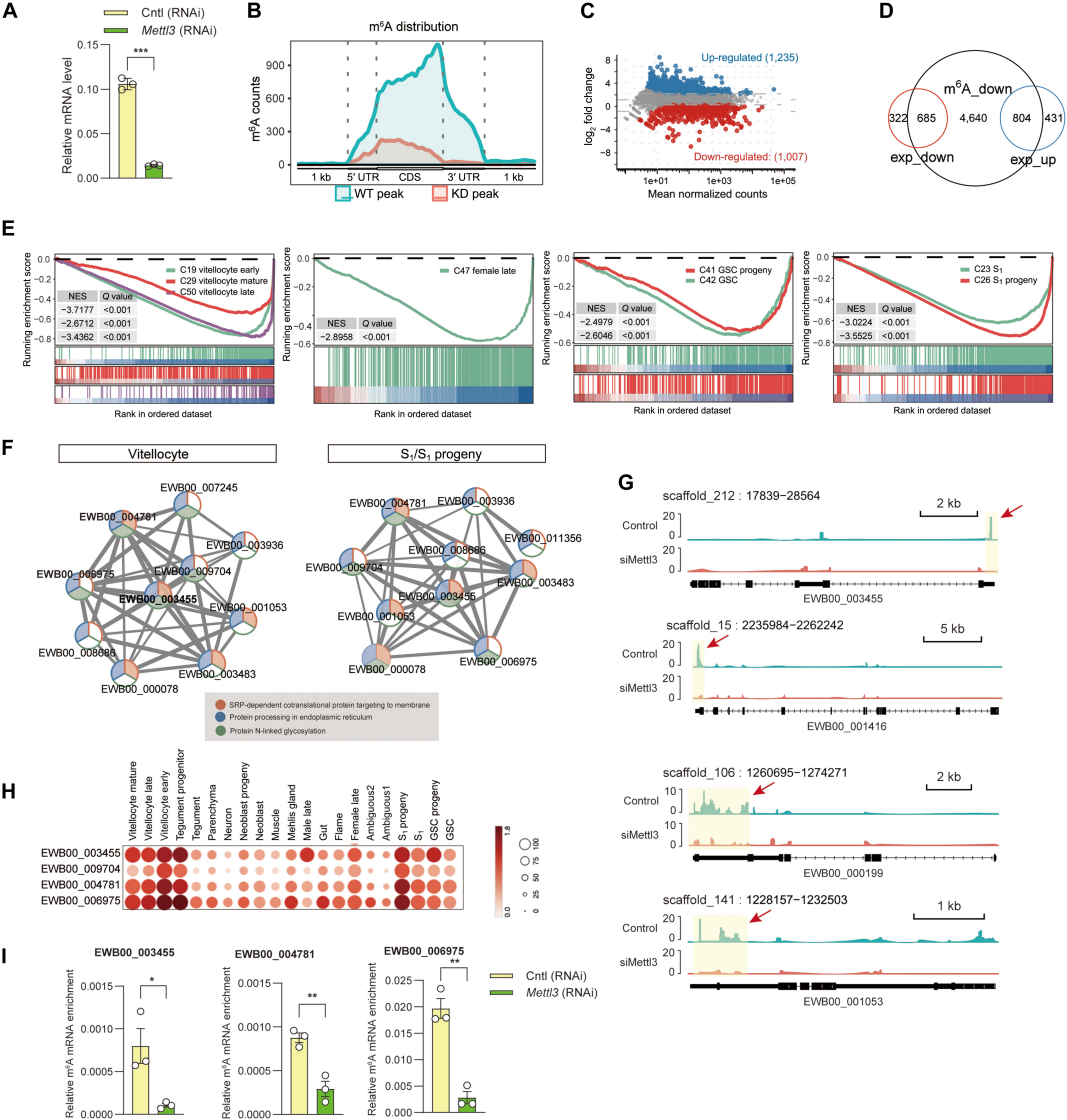
Mechanism of *Mettl3*-mediated N^6^-methyladenosine (m^6^A) modification for regulating female oviposition. (A) Validation of *Mettl3* knockdown (KD) efficiency in female schistosomes via real-time quantitative reverse transcription polymerase chain reaction (RT-qPCR). Data illustrate representative results showing mean ± standard deviation (SD) from an experiment conducted in triplicate. ***P < 0.001. (B) *Mettl3* KD globally decreased the m^6^A level. Peaks were mapped relative to the coding sequence (CDS), 5′ untranslated region (5′ UTR), 3′ untranslated region (3′ UTR), and flanking regions (±1 kb). (C) Differentially expressed genes (DEGs) between *Mettl3*-KD and control worms. Dot plot showing significantly down-regulated genes (red, log_2_FC ≤ −1), up-regulated genes (blue, log_2_FC ≥ 1), and genes with nonsignificant changes (gray). (D) Overlapping of the reduced m^6^A genes and DEGs. Most DEGs exhibited reduced m^6^A levels. (E) Gene set enrichment analysis (GSEA) results indicated that the down-regulated (decreased m^6^A) genes enriched in germline stem cells (GSCs), GSC progeny, female late, vitellocytes (early, mature, and late), S_1_, and S_1_ progeny. (F) Protein–protein interaction (PPI) map networks of the genes exhibiting reduced m^6^A levels in vitellocytes and S_1_/S_1_ progeny. The edges between nodes in the network represent confidence scores, with values ranging from 0.5 to 1.0. (G) Exemplary RNA sequencing (RNA)-seq coverage profiles (m^6^A-immunoprecipitated [m^6^A-IP] vs. input) are shown for EWB00_001416, EWB00_003455, EWB00_000199, and EWB00_001053 with identified m^6^A peaks highlighted. Arrows indicate the target regions. (H) Single-cell RNA sequencing (scRNA-seq) data confirmed the high transcript levels of these vitellocyte PPI network genes within vitellocytes. (I) Independent validation of the reduced m^6^A levels in vitellocyte-associated transcripts via methylated RNA immunoprecipitation (MeRIP)–quantitative reverse transcription PCR (RT-qPCR) in *Mettl3*-KD females. Data illustrate representative results indicating mean and SD obtained from an experiment conducted in triplicate. *P < 0.05; **P < 0.01.

To determine the mechanism by which METTL3-mediated m^6^A modification regulates ovarian development and oviposition, we generated *Mettl3*-KD females (Fig. 5A) and performed MeRIP-seq to assess transcriptome-wide alterations in m^6^A methylation and concomitant changes in gene expressions. *Mettl3* KD resulted in a global reduction of m^6^A signals across the transcriptome (Fig. 5B). Comparative transcriptome analysis identified 2,242 differentially expressed transcripts between *Mettl3*-KD and control groups (1,235 up-regulated and 1,007 down-regulated, *P* < 0.05) (Fig. 5C). Among these, 685 down-regulated and 804 up-regulated genes showed a concurrent decrease in m^6^A levels (Fig. 5D). Gene set enrichment analysis (GSEA) was performed on the differentially expressed genes (DEGs). The results revealed important enrichment in pathways related to female reproduction, including those associated with vitellocytes (early, late, and mature), female late (ovary), GSC/GSC progeny, and S_1_/S_1_ progeny (Fig. 5E and Fig. S6).

Protein–protein interaction (PPI) analysis was performed using the Search Tool for the Retrieval of Interacting Genes/Proteins (STRING), and the results indicated that genes with reduced m^6^A levels formed functional networks enriched in these reproductive cell types (Fig. 5F and Fig. S7). Representative methylated genes within these core networks showed marked reductions in m^6^A levels upon *Mettl3* KD (Fig. 5G), including T-complex protein 1 subunit alpha isoform 1 (EWB00_000199), dolichyl-diphosphooligosaccharide–protein glycosyltransferase subunit 1 (EWB00_003455), nonspecific serine/threonine protein kinase (EWB00_001416), and translocon-associated protein subunit alpha (EWB00_001053). Further analysis of vitellocyte and S_1_/S_1_ progeny-related pathways indicated that these core nodes interact to regulate protein N-linked glycosylation via asparagine (Fig. 5F). Single-cell RNA-seq data confirmed that these genes are highly transcribed in ovarian and vitellocyte populations (Fig. 5H), supporting their functional relevance in reproductive development. The reduction in m^6^A levels for selected core genes in the vitellocyte network was independently validated by MeRIP–RT-qPCR in *Mettl3*-KD females (Fig. 5I). Overall, these findings reveal the molecular basis of METTL3-mediated m^6^A regulation in female oviposition and underscore its essential role in maintaining reproductive competence in *S. japonicum.*

### m^6^A modification maintains male tegument integrity through glutamine metabolism

**Fig. 6.**
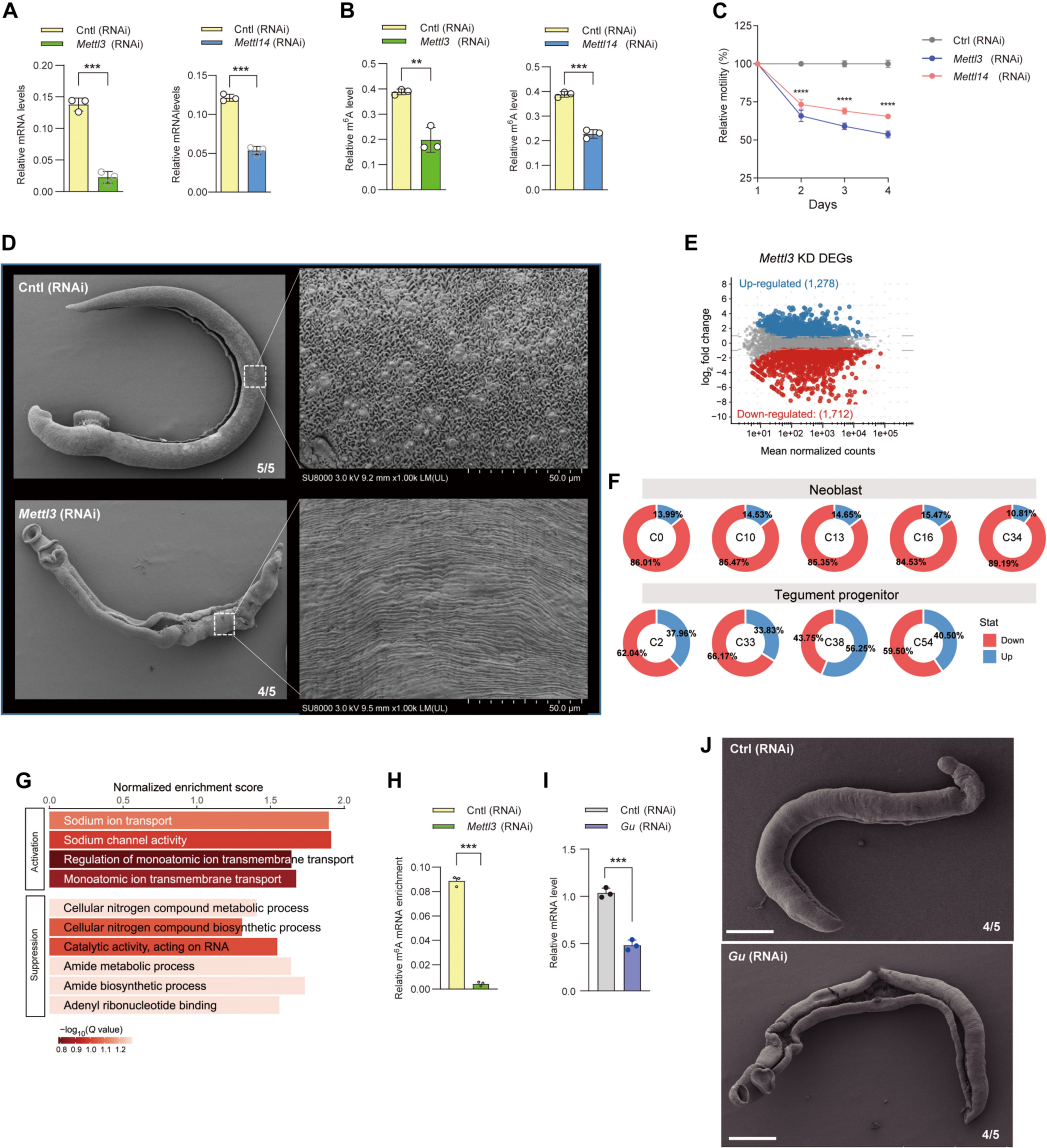
Functional role of *Mettl3*/*Mettl14* in male *Schistosoma japonicum*. (A) Real-time quantitative reverse transcription polymerase chain reaction (RT-qPCR) analyses of the decreased transcript levels of *Mettl3*/*14* in males treated with *Mettl3*/*Mettl14* small interfering RNAs (siRNA). Data are shown as mean ± SD from a representative experiment (*n* = 3). ***P < 0.001. (B) *Mettl3*/*Mettl14* knockdown (KD) decreased the N^6^-methyladenosine (m^6^A) levels in the males. Data are shown as mean ± SD from a representative experiment (*n* = 3). **P < 0.01; ***P < 0.001. (C) *Mettl3*/*14* inhibition led to a decrease in worm motility. Data are representative results observed from 30 worms for each experiment. ***P < 0.001. (D) *Mettl3* suppression leads to morphological defects in male worms. Numbers in the lower right indicate the fraction of worms that are similar to those presented/total number of worms examined. (E) RNA sequencing (RNA-seq) analysis of differential expression genes between *Mettl3*-KD and control worms. Dot plot showing significantly down-regulated genes (red, log_2_FC ≤ −1), up-regulated genes (blue, log_2_FC ≥ 1), and genes with nonsignificant changes (gray). (F) *Mettl3* KD resulted in the down-regulated genes in cell populations, including neoblast and tegument progenitor. (G) Gene Ontology (GO) term enrichment analysis of genes dysregulated following *Mettl3* KD. (H) Quantitative reverse transcription PCR (RT-qPCR) combined with methylated RNA immunoprecipitation (MeRIP) verified the decreased m^6^A levels of glutamine synthetase (EWB00_007982) in *Mettl3*-KD males. Data are shown as mean ± SD from a representative experiment (*n* = 3). ***P < 0.001. (I) Significant reduction of *Glutamine synthetase* transcript levels following RNA interference (RNAi) KD in males. Data are shown as mean ± SD from a representative experiment (*n* = 3). ***P < 0.001. (J) Scanning electron microscopy (SEM) analysis of morphological alterations in male schistosomes after *Glutamine synthetase* KD. Numbers in the lower right indicate the fraction of worms that are similar to those presented/total number of worms examined.

To investigate the functional roles of m^6^A modification in male *S. japonicum*, we inhibited *Mettl3* and *Mettl14* with siRNAs. RT-qPCR confirmed effective KD of both genes compared to controls (Fig. 6A), along with a significant decrease in global m^6^A levels (Fig. 6B). Notably, *Mettl3* suppression severely impaired motility and induced a characteristic body-twisting phenotype (Fig. 6C and Videos S1 and S2). To explore the underlying structural defects, we examined affected males using scanning electron microscopy (SEM), which revealed marked tegumental damage, including the loss of sensory papillae, potentially contributing to the observed motility deficits (Fig. 6D). RNA-seq analysis of *Mettl3*-KD males identified 1,278 up-regulated and 1,712 down-regulated genes (Fig. 6E and Table S4). Integration with single-cell RNA-seq data indicated that the down-regulated genes were mainly enriched in neoblasts, tegumental progenitors, GSCs, and gut lineages (Fig. 6F and Fig. S8), consistent with the observation of m^6^A enrichment in testes and teguments (Fig. 6E). Further analysis of GO indicated that these down-regulated genes were associated with catalytic activity and amide metabolism, while up-regulated genes were involved in monoatomic ion transmembrane transport (Fig. 6G and Table S5). Among the affected transcripts, glutamine synthetase (GS; EWB00_007982) (Fig. S9), a highly m^6^A-modified gene, showed a significant reduction of m^6^A modification in *Mettl3* KD, as validated by MeRIP–RT-qPCR (Fig. 6H). Given that GS supplies nitrogen for purine/pyrimidine synthesis and amino acid production [33], we asked whether its dysregulation could partially explain the phenotypic consequences of *Mettl3* suppression. Indeed, siRNA-mediated inhibition of *GS* (Fig. 6I) resulted in a screwlike body morphology and general tissue damage (Fig. 6J), closely resembling the defects observed in *Mettl3*-KD males. Collectively, these results demonstrate that loss of the m^6^A writer METTL3 disrupts the methylation of metabolism-related transcripts such as GS, which in turn compromises tegumental integrity and body architecture in male schistosomes, ultimately impairing motility and survival.

## Discussion

**Fig. 7.**
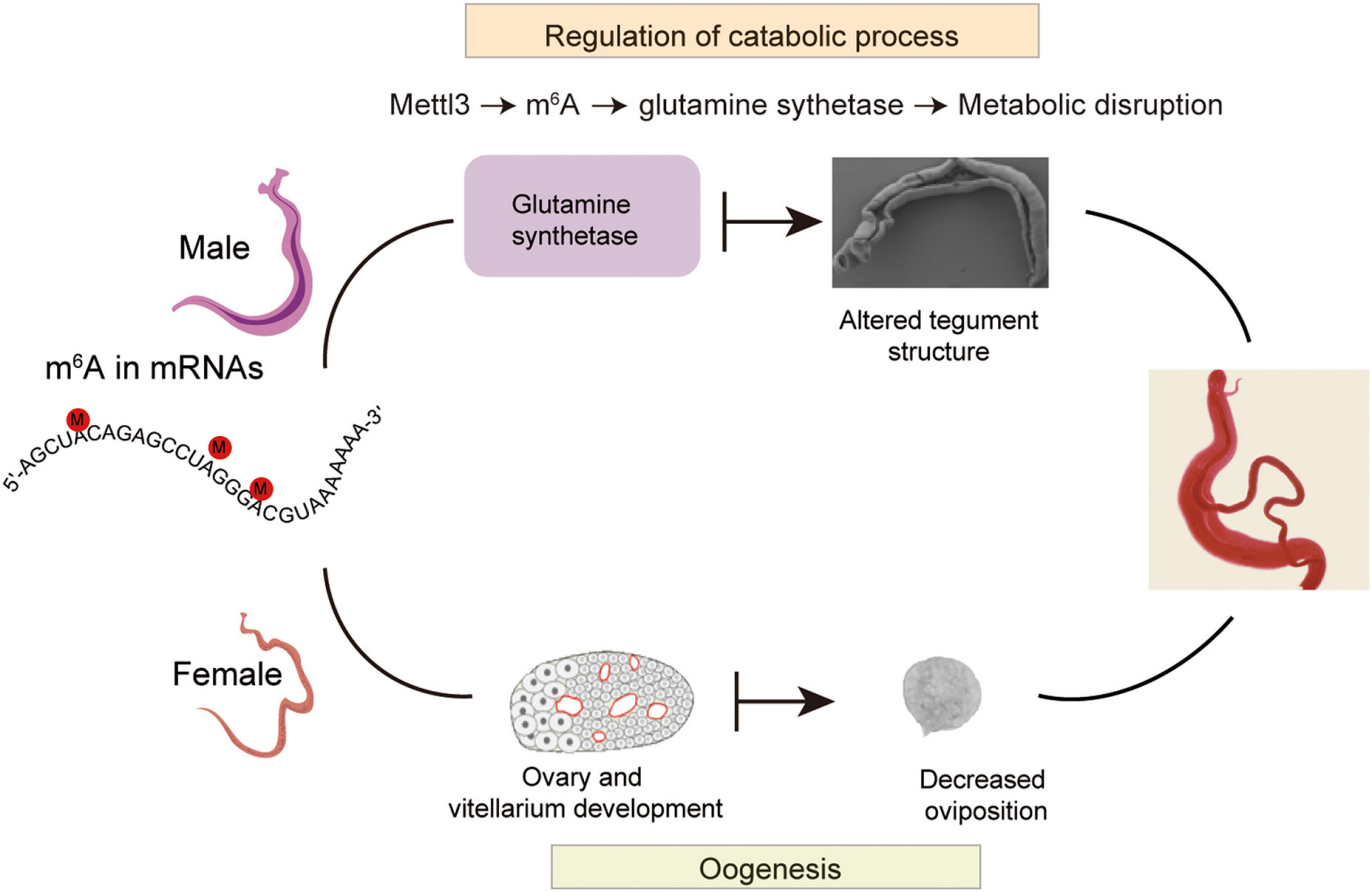
The proposed model for N^6^-methyladenosine (m^6^A)-mediated regulation of reproductive development and oviposition in females and metabolism in males of *Schistosoma japonicum*.

In the present study, we delineate the essential roles of m^6^A mRNA modification in *S. japonicum*. We generated high-resolution, quantitative m^6^A maps for adult males and females, which revealed distinct sex-specific methylation landscapes. Our findings demonstrate that m^6^A modification is essential for regulating female reproductive development and oviposition, as well as for maintaining male tegument integrity (Fig. 7). Importantly, functional analysis showed that suppression of the m^6^A writer *Mettl3* significantly reduced m^6^A levels and impaired normal development in both sexes. These results provide the first functional evidence for m^6^A-mediated epitranscriptomic regulation in a pathogenic helminth.

Schistosomes are unique among flatworms in being dioecious. Morphologically, the male worms are large and muscular, with a specialized groove along their ventral surface, the gynecophoral canal, and the male clasps the female to allow female development. Previous studies reported a gender-specific dynamic transcriptome, which hints at the division of labor in males and females [34,35]. Unlike male schistosomes, females are committed to egg production. The ovary produces oocytes, while the vitellaria produce vitellocytes. The vitellocytes provide both eggshell constituents and nutrition for the developing embryo [35]. In adult females, genes related to egg production and hemoglobin digestion are markedly enriched compared to those in immature females [36]. GO analysis of m^6^A-modified genes in females revealed their association with vitellocytes and oogenesis, and suppression of *Mettl3* showed that morphologic alterations of gonads led to decreased oviposition, reduced ovary proliferating cells in the ovary, and altered egg morphology. These results demonstrated that m^6^A modification sustains reproductive capacity in female *S. japonicum*.

To gain insight into m^6^A-modified mRNAs for regulating egg production, we constructed a PPI network based on differentiated expressed genes after *Mettl3* suppression in female *S. japonicum*. By focusing on the genes enriched in vitellocytes (early, late, and mature) and female late (ovary), we found that the core nodes of the interaction network (EWB00_003455, EWB00_009704, EWB00_004781, and EWB00_006975) involve protein N-linked glycosylation via asparagine. Among them, EWB00_003455 encodes dolichyl-diphosphooligosaccharide–protein glycosyltransferase subunit 1, which plays an important role in protein translocation across the endoplasmic reticulum [37]. Taken together, these results suggested that protein modification and translocation could be essential role in m^6^A-modification-mediated egg production in female *S. japonicum*.

In contrast to female worms, genes associated with tegument structure and movement were more enriched in adult males [38,39], which corroborates our present observations. The male tegument is vital during copulation, as the male uses the tegument to grasp females within the gynecophoral canal, an essential process for female sexual maturation and egg production [40]. It also serves as a platform for the absorption of substrates such as glucose, amino acids, cholesterol, and lipids [41,42] and helps in osmoregulation and excretion [43]. We observed that *Mettl3* inhibition suppresses dysregulated metabolic transcripts such as those of the cellular nitrogen compound metabolic process, cellular nitrogen compound biosynthetic process, amide metabolic process, and amide biosynthetic process. By focusing on nitrogen metabolism, we noted that *Mettl3* inhibition led to decreased transcript levels of *GS* (EWB00_007982) and increased transcript levels of glutamate transporter (EWB00_006708). A previous study indicated that GS was mainly distributed on the tegument and parenchyma in adult worms [33]. Another study suggested that a high-affinity glutamate transporter on the schistosome muscle membrane played a critical role in muscle contraction in *S. mansoni* [44]. Given that substantial phenotypes including severe tegumental damage and body twisting observed in either *Mettl3*-inhibited or *GS*-suppressed male worms, our findings highlighted the important roles of m^6^A modification in males for maintaining male tegumental integrity that is required for metabolic homeostasis, particularly GS-mediated nitrogen metabolism.

m^6^A RNA modification is a conserved mechanism [22,45] that posttranscriptionally regulates gene expression in eukaryotes, with emerging roles in pathogen biology and virulence [12,46]. In parasites, most studies focused on protozoan *P. falciparum*; in a pioneering study, m^6^A modifications were identified and shown to be regulated by an m^6^A methyltransferase (PfMT-A70) [15]. Then, 2 YTH domain proteins were demonstrated to bind to m^6^A-containing mRNA for regulating protein translation [16,17]. Recently, m^6^A in poly(A) tails was found to stabilize the transcripts of variant surface glycoproteins by regulating mRNA degradation in *T. brucei* [12]. Moreover, several m^6^A interactors such as METTL3, WTAP, or YTH1 were shown to modify mRNA 3′ ends for regulating parasite viability in *T. gondii* [18]. However, the related study in pathogen helminths is not reported so far. Our study provides the first evidence that m^6^A serves a previously unrecognized yet pivotal role in a pathogenic helminth, where it is indispensable for female reproductive development and parasite survival in *S. japonicum*. Our work expands the knowledge of epitranscriptomic regulation in helminths and identifies the m^6^A machinery as a potential target for therapeutic intervention against schistosomiasis.

## Materials and Methods

### Parasite culture

*S. japonicum* (Anhui isolate) cercariae were obtained from the intermediate host *Oncomelania hupensis* through the Centre of National Institute of Parasitic Disease (Chinese Center for Disease Control and Prevention, Shanghai, China). Mice were infected with 50 to 150 *S. japonicum* cercariae via abdominal skin penetration. New Zealand rabbits received percutaneous infection with approximately 1,000 to 1,500 *S. japonicum* cercariae. Schistosomes were collected at the indicated date, washed with phosphate-buffered saline (PBS), and used for subsequent experiments. The animal experiments were approved by the Institution Animal Ethics Committee, Tongji University (TJAA00621101).

### m^6^A dot blot assay

Total RNAs were isolated from adult male and female *S. japonicum* (28 dpi) using the TRIzol reagent (Thermo Fisher Scientific, Waltham, MA, USA). RNA concentrations of 1, 0.5, and 0.25 μg were spotted onto an Amersham Hybond-N+ membrane optimized for nucleic acid transfer (GE Healthcare, Chicago, IL, USA). Following air-drying, the membrane containing RNAs was subjected to ultraviolet cross-linking in HB-1000 HybriLinker (UVP, Upland, CA, USA). The membrane was then blocked with a 5% nonfat milk solution in PBS with Tween 20 (PBST) for 2 h, followed by overnight incubation at 4 °C with an anti-m^6^A antibody (ab208577, Abcam) diluted 1:1,000 in the blocking buffer. After 3 consecutive washes with PBST, the membranes were incubated with a horseradish peroxidase (HRP)-conjugated anti-rabbit immunoglobulin G (IgG) secondary antibody (1:5,000 dilution) for 1 h at room temperature. Finally, chemiluminescent detection was performed using ECL Prime Blotting Detection Reagent (GE Healthcare) with visualization in a chemiluminescence imaging system. RNA oligonucleotides modified with m^6^A and their unmodified counterparts served as positive and negative controls, respectively.

### Identification of mRNA modifications by LC–MS/MS

Total RNAs were isolated from 28-dpi male and female *S. japonicum* using TRIzol reagent (Thermo Fisher Scientific), and the product was subjected to DNase I (Thermo Fisher Scientific) treatment. mRNA was purified from the total RNA preparations using a Dynabeads mRNA purification kit (Ambion, Texas, USA), and RNA integrity was evaluated via Agilent Bioanalyzer 2100 (Agilent Technologies, Palo Alto, CA). mRNA was enzymatically fragmented to single nucleotides, and the pre-treated nucleoside solution was subjected to deproteinization using a Sartorius 10,000-Da molecular weight cutoff spin filter. Analysis of nucleoside mixtures was conducted employing a ultra-performance liquid chromatography–electrospray ionization–tandem mass spectrometry system, consisting of an ExionLC AD liquid chromatograph and an Applied Biosystems 6500 Triple Quadrupole mass spectrometer as described previously [47]. Samples were chromatographed on an AB SCIEX QTRAP 6500+ column (AB Sciex LLC, Framingham, USA; 1.8 μm, 2.1 mm ∗ 100 mm). The column effluent was directed to an electrospray ionization triple quadrupole-linear ion trap (QTRAP), liquid chromatography–tandem mass spectrometry, which was equipped with an ESI Turbo Ion-Spray interface. This system operated in both positive and negative ion modes and was controlled by the Analyst 1.6.3 software (AB Sciex, Framingham, MA, USA) using the following parameters: ion source, turbo spray; source temperature, 550 °C; and ion spray voltage, 5,500 V. Peak area quantification and data processing were performed using the Analyst 1.6.3 software.

### MeRIP sequencing

At 28 dpi, parasitic worms were harvested from infected rabbits and manually separated into males and females. The total RNA of females and males was isolated for MeRIP (3 biological replicates). m^6^A MeRIP was performed following previously described m^6^A sequencing methodologies with minor modifications [48]. Briefly, RNA (30 μg for each sex) was fragmented using fragmentation buffer. Fragmented RNA was incubated for 2 h at 4 °C with m^6^A antibody from the GenSeq m^6^A-Me-RIP Kit (GenSeq Inc., China). Next, the input and immunoprecipitation (IP) products were used for RNA-seq library construction via the NEBNext Ultra II Directional RNA Library Prep Kit (New England Biolabs, USA).

Both the input samples without IP and the m^6^A-IP samples were used. Library quality assessment was conducted using the Bioanalyzer 2100 system (Agilent Technologies, USA), and the qualified libraries were then subjected to high-throughput sequencing on an Illumina HiSeq platform, with 150-bp paired-end reads.

### Bioinformatic analyses of MeRIP-seq

For as well as quality control, the MeRIP-seq data were first subjected to adaptor trimming using trim_galore software (version 0.6.10, https://github.com/FelixKrueger/TrimGalore) [49] with the following settings (-q 25 --paired --length 50 --trim-n). Then, the obtained paired-end reads were aligned to the *S. japonicum* reference genome (Assembly PRJNA520774) [50] using STAR (version 2.7.11a) [51] with the settings (--outFilterMultimapNmax 30 --genomeLoad NoSharedMemory --outFilterMismatchNmax 999 --alignIntronMin 20 --alignIntronMax 1000000 --outFilterMismatchNoverReadLmax 0.04 --alignMatesGapMax 1000000 --sjdbOverhang 140). Subsequently, reads that mapped to ribosomal RNA excluded. Statistically significant m^6^A peaks in each sample were identified using exomePeak2 (version 1.10.0) [52] with the parameters strandness = “unstrand”, fragment_length = 100, p_cutoff = 1e–10, mode = “exon”, bin_size = 25, step_size = 25, motif_based = TRUE, and motif_sequence = “NNNNNANNNNN”. Significant peaks were then visualized utilizing the GuitarPlot function of the R package Guitar (version 2.18.0) [53].

### Comparative analyses of female and male MeRIP-seq results

Following alignment, MeRIP sequencing data were analyzed using the multiBamSummary tool from deepTools (version 3.5.4) with the BED-file option to generate principal component analysis results via plotPCA. Venn diagrams depicting m6A peaks identified by exomePeak2 across different replicates were constructed using the euler function from the eulerr (version 7.0.2) package [54]. The abundance of m^6^A modifications per gene was defined as the average log_2_FC of all m^6^A peaks within that gene, and visualized using the pheatmap function from the pheatmap (version 1.0.12) package (https://github.com/raivokolde/pheatmap).

### GO analysis

The genome of *S. japonicum* was functionally annotated using the online version of eggnog-mapper (http://eggnog-mapper.embl.de/) [55]. Enrichment analysis was carried out using the R package clusterProfiler, with the settings of pAdjustMethod = “BH” and qvalueCutoff = 0.2.

### Motif analysis

The fasta sequences of significant m^6^A peaks were stacked centered on the A residue and visualized using the R package motifStack (version 1.46.0) [56]. To further validate the motifs, we also utilized the online version of MEME-ChIP (https://meme-suite.org/meme/tools/meme-chip) to directly perform motif discovery [57].

### MeRIP–RT-qPCR

Total RNAs from adult male and female *S. japonicum* (28 dpi) were isolated using TRIzol reagent. IP was performed following the protocol described in the previous sections on MeRIP-seq. Equal aliquots of eluted RNA underwent reverse transcription into complementary DNA using PrimeScript RT Reagent Kit (TaKaRa, Shiga, Japan), followed by RT-qPCR analysis using the SYBR Premix Ex Taq kit (TaKaRa) following the manufacturer’s instructions. The PCR amplification program consisted of an initial denaturation step at 95 °C lasting 2 min, followed by 40 cycles each comprising denaturation at 95 °C for 20 s, annealing at 55 °C for 20 s, and extension at 72 °C for 20 s. IP efficiency was normalized by comparing the expression to the input samples. All reactions were run in triplicate, and relative expression was determined using the 2^−ΔCt^ method [58]. The primers are listed in Table S1.

### Orthologous and phylogenetic analyses of the factors related to m^6^A modification

To identify candidate proteins associated with m^6^A factors in *S. japonicum*, we conducted a blastp search (version 2.12.0+) against the *S. japonicum* proteome, using known components of the m^6^A writer complex from other eukaryotic organisms as query sequences [59]. Protein sequence alignments were carried out with the MEGA software (version 11.0.13) [60], and the resulting alignments were visualized using GeneDOC (version 2.7) [61] and Geneious (version 2024.0). Phylogenetic tree construction was performed in MEGA with 2,000 bootstrap replications, utilizing the best-fit substitution model selected based on the lowest Bayesian information criterion value. The resulting phylogenetic trees were visualized and annotated using Evolview (v3) [62].

### RT-qPCR analyses of the transcript levels of m^6^A writers in different parts of dissected worms

To evaluate the expression levels of m^6^A writers across various anatomical regions of adult male and female worms, female worms (28 dpi) were surgically separated into 3 distinct segments: the anterior region, ovary-enriched section, and vitellarium-enriched segment. In parallel, adult males (28 dpi) were dissected into 2 primary parts: the testis-enriched region and the posterior segment. The dissected samples were subjected to RNA isolation for determining the transcript levels of m^6^A writers by qPCR as described above.

### WISH analyses of m^6^A writers

To generate probes, the T7 promoter sequence was incorporated into specific primers of m^6^A writer genes (Table S1). Subsequent purification of the PCR amplicons was performed using the GeneJET PCR Purification Kit (Thermo Scientific). Next, digoxigenin (DIG)-labeled probes were synthesized using the MEGAscript Kit (Thermo Scientific).

WISH analyses were carried out according to previous publications with minor modifications [63,64]. Briefly, worms (28 dpi, at least 20 worms for each sex) were fixed for 15 min in a 4% paraformaldehyde (PFA) solution prepared with PBS containing 0.3% Triton X-100. Worms were subjected to a reduction step, incubated for 5 to 10 min at 37 °C in a solution composed of 50 mM dithiothreitol, 1% NP-40, and 0.1% sodium dodecyl sulfate, followed by a dehydration series using methanol gradients (100% PBSTx [PBS with Triton X-100], 50% methanol, and 100% methanol), with each step lasting 5 min. Subsequently, the parasites were bleached overnight under light exposure in a 5% hydrogen peroxide solution diluted in methanol. After bleaching, the worms were rinsed with methanol and transferred to PBSTx. For permeabilization, they were treated with proteinase K at a concentration of 1 μg/ml in PBS for 15 to 20 min, followed by rinsing with PBSTx. Prehybridization began with incubation in hybridization buffer (50% deionized formamide, 1.3× saline-sodium citrate buffer [SSC] pH = 5.5, 5 mM EDTA, 50 μg/μl RNA, and 0.2% Tween) mixed with PBSTx (1:1) for 10 min. After washing at 57 °C, fresh buffer was added for 2 h. Prewarmed probes (5 μM) were added and incubated overnight at 57 °C. Washing used preheated solutions: prehybridization solution with 2× SSC (1:1), 2× SSC with Triton X-100, and 0.2 × SSC with Triton X-100. Each washing procedure was performed 2 × 30 min at 58 °C. After cooling to room temperature, the worms were washed twice using maleic acid buffer (100 mM maleic acid, 0.1% Tween-20, 150 mM NaCl, pH 7.5).

The schistosomes were immersed in 10% horse serum solution for 2 h, followed by incubation at 4 °C overnight with an anti-digoxigenin-alkaline phosphatase (anti-DIG-AP; with dilution of 1:2,000) conjugated antibody (Roche). The schistosomes underwent a 2-h wash in maleic acid buffer, replaced every 20 min. The worms were then subjected to a 10-min incubation in AP buffer (100 mM Tris, pH 9.5; 100 mM NaCl; 0.1% Tween-20 in 10% polyvinyl alcohol; 50 mM MgCl_2_) and a 3-h staining in NBT/BCIP solution (Roche). After washing with PBST and postfixing in 4% PFA, the worms were mounted in 80% glycerol beneath glass coverslips and visualized using a light microscope (Olympus, Japan).

### Immunofluorescence staining

At 28 dpi, adult worms were isolated and transferred to 2-ml Eppendorf tubes and immediately fixed in 4% PFA in PBSTx overnight. The following day, the worms (25 to 30 worms for each sex) underwent permeabilization and blocking procedures using proteinase K (5 μg/ml) and 5% bovine serum albumin at room temperature for 30 min. Following a brief washing with PBSTx, worms were probed using m^6^A primary antibody (with a dilution of 1:1,000) overnight at 4 °C and then incubated with appropriate secondary antibody (Alexa Fluor 594, with a dilution of 1:5,000) and 4′,6-diamidino-2-phenylindole (DAPI; 1 μg/ml) for 1 h. After a brief washing, the worms were temporarily mounted in Vectashield (Beyotime Biotechnology, China) and were visualized using LSM 780 confocal laser microscopy (Carl Zeiss, Oberkochen, Germany).

### Single-cell RNA-seq analyses

The expression profiles of selected genes at different cell clusters were determined by using the datasets from an independent study (doi: https://doi.org/10.64898/2026.01.20.700708).

### Worm culture, RNA interference, and Western blot analyses

At 24 dpi, adult worms were harvested from infected mice and cultured in a 12-well plate containing 2 ml of complete RPMI 1640 medium supplemented with 15% heat-inactivated fetal bovine serum and 5% penicillin/streptomycin (10,000 units of penicillin and 10 mg/streptomycin) within a 5% CO_2_ incubator at 37 °C following the protocol described previously [65]. Briefly, cultured worms were electroporated (BTX, MA, USA) with 3 μg of gene-specific and control siRNA (synthesized by Shanghai GenePharma, Shanghai, China) at 125 V for 20 ms and one pulse in 200 μl RPMI 1640 medium. Then, worms were transferred to 12-well cell plates containing 2 ml of fresh culture medium. After 4 d post-transfection, the worms were subjected to RNA quantification by qPCR as described above to evaluate the inhibition effect, and a part of the worms was analyzed by SEM.

Western blot analyses were performed as described previously [3]. The primary antibodies against METTL3 (Cat. No.: 15073-1-AP, 1:5,000 dilutions) (Proteintech, China) and anti-beta actin (Cat. No.: 66009-1-Ig, 1:5,000 dilutions) (Proteintech, China) were used. HRP Conjugated Goat Anti-Mouse IgG was used as the second antibodies (1:5,000 dilutions, Cat. No.: CW0102S, CoWin Biosciences, China).

### m^6^A quantification

The EpiQuik m^6^A RNA Methylation Quantification Kit (EpigenTek, Farmingdale, NYA, USA; P-9005-96) was used to determine the level of m^6^A methylation in different stages, including egg, cercariae, 14 d, 21 d, and 28 d and *Mettl3*/*14*-suppressed worms. Eggs were harvested from the liver of infected rabbits, and cercariae were collected from positive O. hupensis (Chinese Center for Disease Control and Prevention, Shanghai, China). Relative m^6^A levels were quantified calorimetrically at a wavelength of 450 nm. All experiments were performed in triplicate.

### EdU staining in female ovaries

At 96 h posttreatment (*Mettl3* siRNA and control siRNA), the worms were collected, and 20 μM EdU (Click-iT Plus EdU Cell Proliferation Kit; Invitrogen, Waltham, USA) was added to the culture media in accordance with the manufacturer’s instructions. The worms underwent washing with PBSTx, dehydrated with increasing grades of methanol in PBSTx for 10 min. Fresh 100% methanol was introduced, and the specimens were stored at −20 °C. For rehydration, the worms were placed in 50% methanol in PBSTx for 10 min, followed by incubation in PBSTx alone. Permeabilization was achieved by treating the worms with proteinase K for 30 min, after which they were fixed using 4% formaldehyde in PBSTx. The worms were then incubated with 500 μl of Click-iT EdU reaction buffer for 30 min. After washing using PBS and incubating with DAPI overnight at 4 °C, the worms were mounted in Vectashield (Beyotime Biotechnology, China). The images were captured using a Nikon A1R confocal microscope (Nikon, Melville, NY, USA) with excitation wavelengths set at 488 nm for Alexa Fluor 488 and 358 nm for DAPI.

### Ovarian morphology by confocal microscopy

At 96 h posttreatment, the female worms were fixed in AFA (containing 48% alcohol, 25% formalin, and 2% acetic acid) and processed for staining with carmine, following a previously described protocol [66]. Parasite samples underwent clearing with xylene before mounting in DPX medium (a mixture of distyrene, plasticizer, and xylene) in preparation for confocal microscopic examination. After adequate drying, the worms were subjected to morphological analysis utilizing a Nikon CLSI laser confocal microscope equipped with a 488-nm He/Ne laser.

### Fast Blue BB staining

At 96 h following treatment, female worms were fixed for 4 h in PBSTx containing 4% formaldehyde. Subsequently, they were incubated with a 10 mg/ml solution of Fast Blue BB (Thermo Scientific, USA) prepared in PBSTx for 5 min at room temperature. After development, the samples were washed 3 times with PBSTx 3 times. To evaluate morphological alterations in the female reproductive system, the parasites were cleared using 80% glycerol and then mounted onto glass slides for microscopic examination.

### Egg count

Following *Mettl3* siRNA and control siRNA treatment, at 24-h intervals, the eggs in each well were collected by centrifuging the medium. The culture medium was decanted and washed with PBS (pH 7.4). Then, the pellet containing worm eggs was resuspended gently in PBS and transferred to a glass slide, and the eggs were counted under a light microscope (Olympus).

### Molecular docking

Molecular docking between sjMETTL3 (EWB00_007032) and the small-molecule inhibitor STM2457 was performed using Molecular Operating Environment (MOE). The London dG scoring function was employed, with the calculation protocol as described previously [67]. The top-ranked docking pose was ultimately selected for subsequent visualization and analysis.

### *Mettl3* inhibitor treatment

*S. japonicum* were collected from infected mice at 28 dpi, and females were separated and 10 worms were cultured in each well of 12-well flat-bottom plates as described previously [64]. STM2457 (MCE) dissolved in dimethyl sulfoxide was added to the culture medium at the indicated concentrations. The number of eggs produced was counted during the time course.

### In vivo suppression of *SjMettl3* in mice infected with *S. japonicum*

BALB/c mice were obtained from the Hangzhou QIZHEN Animal Co., Ltd. Mice (*n* = 15) were randomly allocated into 3 groups. Each mouse was infected with *S. japonicum* cercariae (*n* = 80 ± 5) via abdominal skin penetration. Following a previous injection protocol [68], at 20 dpi, the mice in each group received a tail vein injection of 1 OD *SjMettl3* siRNA or control siRNA (chemically synthesized by Generay Biotechnology [Shanghai, China]) by dilution in 100 μl of nuclease-free H_2_O as PBS. At days 23, 26, and 29 dpi, 3 additional injections were administrated. At 35 dpi, worms were collected through liver perfusion. Egg deposition in the liver was counted. Liver tissue was fixed and analyzed for hematoxylin and eosin staining as described previously [69].

### MeRIP analysis of *mettl3*-KD females

Adult worms (24 dpi) were collected from infected mice, male and female worms were manually separated, and females were cultured in 12 well flat-bottom culture plates containing 2 ml of RPMI 1640 culture medium with 15% fetal bovine serum (Gibco) and 5% penicillin/streptomycin (10,000 U of penicillin and 10 mg of streptomycin in 0.9% NaCl, Gibco). *Mettl3* and control siRNA were electroporated into cultured worms (125 V, 20 ms, 1 pulse in 200 μl RPMI 1640 medium). At 96 h post-electroporation, one-part worms was collected for RNA isolation for MeRIP-seq as described above and the rest of the worms were subjected to RT-qPCR analysis. The siRNA sequences are listed in Table S1.

### *Mettl3*-KD females and MeRIP-seq data analyses

At 96 h of post-electroporation, worms were collected for RNA isolation for MeRIP-seq as described above. The procedures for read trimming, alignment, and peak calling were performed as described above. Genes showing an overall reduction in total methylation levels—measured by a decrease in the summed diff.log2FC of all m^6^A peaks per gene—were identified as having reduced m^6^A levels.

### *Mettl3*-KD RNA-seq data analyses

The procedures for read trimming and mapping were performed as described in MeRIP-seq analysis. Read counts were then obtained using featureCounts (version 2.0.6). DEG analysis was performed using the Bioconductor package DESeq2 (version 1.42.1), and DEGs were defined based on statistical significance (false discovery rate < 0.05) and fold change > 2.

### Gene set enrichment analysis

GSEA was carried out utilizing the GSEA of the clusterProfiler R package [70]. Several sets of *S. japonicum* single-cell genes (vitellocyte, S_1_/S_1_ progeny, female late, and GSC/GSC progeny) were selected for enrichment analysis. Following the GSEA guidelines (https://www.gsea-msigdb.org/), terms with an adjusted *P* threshold of ≤0.25 (*P* adjusted ≤ 0.25) were retained for further analysis. The significantly enriched terms were subsequently visualized using the gseaNb function of the GseaVis R package (version 0.1.0) [71].

### Protein interaction network analysis

The genes showing both significantly differentially expressed and decreased m^6^A levels upon *mettl3* KD were focused on PPI network analysis. Interactions with a confidence score of ≥0.5 (minimum required interaction score) were retained to construct the network. Subsequent analysis was performed in Cytoscape (version 3.10.3) [72] using the cytoHubba plug-in (version 0.1) [73]. To identify the most central proteins within the network, the maximal clique centrality (MCC) algorithm was employed using cytoHubba [73], and the top 10 hub genes were selected based on their MCC scores.

### *Mettl3*/*Mettl14* KD in males

*Mettl3*/*Mettl14* siRNA and control siRNAs (3 μg per experiment, Shanghai GenePharma) were delivered into adult males (24 dpi) by electroporation (125 V, 20 ms, 1 pulse in 200 μl of RPMI 1640 medium) as described above. At 96 h post-electroporation, a part of worms was collected for RNA-seq analysis, a part of worms was collected for RT-qPCR analysis and m^6^A quantification as described above, and a part of worms were analyzed by SEM. The siRNA sequence and primers are provided in Table S1.

### MeRIP–RT-qPCR analysis of *Mettl3*-KD females/males

At 96 h posttreatment, *Mettl3*-siRNA-suppressed females/males and control-siRNA-treated females/males were collected. Then, the total RNAs were isolated. MeRIP was carried out as described above, and the elution was subjected to RNA isolation for qPCR analyses.

### Worm motility

Following *Mettl3*/*Mettl14* KD in male worms, worm motility, including active movement and slow movement, was observed under the microscope (Olympus) at 24-h intervals. The KD parasite was compared to the control worms.

### Suppression of *glutamine synthetase* in males

*GS*-specific and control siRNAs (3 μg per experiment, Shanghai GenePharma) were delivered into adult males (24 dpi) by electroporation (125 V, 20 ms, 1 pulse) in 200 μl of RPMI 1640 medium. Following electroporation, worms were transferred into 12-well plates containing fresh media. At 96 h post-electroporation, a part of the worms was collected for RNA quantification as described above, a part of the worms was collected for MeRIP–RT-qPCR as described above, and a part of the worms was analyzed by SEM. The siRNA sequence and primers are listed in Table S1.

### Scanning electron microscopy

Four days following electroporation, the worms were harvested and rinsed thrice with PBS (pH 7.4), and then were fixed with 4% PFA and 2.5% glutaraldehyde (System Biosciences, Palo Alto, CA) at 4 °C for 2 d. Postfixation was carried out with 1% osmium tetroxide (OsO_4_) (System Biosciences) at ambient temperature, followed by dehydration through a gradient acetone series (TiTan, Shanghai, China). For SEM analysis, specimens underwent freeze-drying and were coated with platinum using a PMC-500 multicoater (Mejwafosis, Tokyo, Japan). Images were acquired using JEOL JSM-6380LV at 2 to 10 kV (JEOL Ltd., Tokyo, Japan).

### Statistical analysis

Statistical analyses were performed using the R language (version 4.3.3; R Core Team, 2024). For analyzing differential gene expression in RNA-seq datasets, negative binomial generalized linear models were employed, with significance determined via the Wald test. Comparative analyzes were performed by the Student *t* test or one-way analysis of variance. Statistical significance is indicated as follows: *P < 0.05, **P < 0.01, and ***P < 0.001.

## Data Availability

The raw data involved in this study have been deposited into the Gene Expression Omnibus the GenBank database with accession nos. GSE306704 and GSE306705 under the BioProjects PRJNA1311845 and PRJNA1311846. The code for main analyses is openly available on GitHub (https://github.com/Alex-zzh/SJ_analysis) under the MIT License. Custom-built R packages and essential datasets are archived on Zenodo (https://doi.org/10.5281/zenodo.18427709).

## Supplementary Materials

Supplementary 1 Figs. S1 to S9 Tables S1 to S5 Videos S1 and S2

## References

[1] LoVerde PT. Schistosomiasis. Adv Exp Med Biol. 2019;1154:45–70. 31297759 10.1007/978-3-030-18616-6_3

[2] Colley DG, Bustinduy AL, Secor WE, King CH. Human schistosomiasis. Lancet. 2014;383(9936):2253–2264. 24698483 10.1016/S0140-6736(13)61949-2 PMC4672382

[3] Liu J, Giri BR, Chen Y, Luo R, Xia T, Grevelding CG, Cheng G. *Schistosoma japonicum* IAP and Teg20 safeguard tegumental integrity by inhibiting cellular apoptosis. PLoS Negl Trop Dis. 2018;12(7): Article e0006654. 30044778 10.1371/journal.pntd.0006654 PMC6078320

[4] Pearce EJ, MacDonald AS. The immunobiology of schistosomiasis. Nat Rev Immunol. 2002;2(7):499–511. 12094224 10.1038/nri843

[5] Zhou KI, Shi H, Lyu R, Wylder AC, Matuszek Ż, Pan JN, He C, Parisien M, Pan T. Regulation of co-transcriptional pre-mRNA splicing by m^6^A through the low-complexity protein hnRNPG. Mol Cell. 2019;76(1):70–81 e79. 31445886 10.1016/j.molcel.2019.07.005 PMC6778029

[6] Chen RX, Chen X, Xia LP, Zhang JX, Pan ZZ, Ma XD, Han K, Chen JW, Judde JG, Deas O, et al. N^6^-methyladenosine modification of circNSUN2 facilitates cytoplasmic export and stabilizes HMGA2 to promote colorectal liver metastasis. Nat Commun. 2019;10(1):4695. 31619685 10.1038/s41467-019-12651-2 PMC6795808

[7] Shan T, Liu F, Wen M, Chen Z, Li S, Wang Y, Cheng H, Zhou Y. m^6^A modification negatively regulates translation by switching mRNA from polysome to P-body via IGF2BP3. Mol Cell. 2023;83(24):4494–4508.e6. 38016476 10.1016/j.molcel.2023.10.040

[8] Meyer KD, Jaffrey SR. Rethinking m^6^A readers, writers, and erasers. Annu Rev Cell Dev Biol. 2017;33:319–342. 28759256 10.1146/annurev-cellbio-100616-060758 PMC5963928

[9] Dominissini D, Moshitch-Moshkovitz S, Schwartz S, Salmon-Divon M, Ungar L, Osenberg S, Cesarkas K, Jacob-Hirsch J, Amariglio N, Kupiec M, et al. Topology of the human and mouse m^6^A RNA methylomes revealed by m^6^A-seq. Nature. 2012;485(7397):201–206. 22575960 10.1038/nature11112

[10] Roundtree IA, Evans ME, Pan T, He C. Dynamic RNA modifications in gene expression regulation. Cell. 2017;169(7):1187–1200. 28622506 10.1016/j.cell.2017.05.045 PMC5657247

[11] Khan FA, Nsengimana B, Awan UA, Ji XY, Ji S, Dong J. Regulatory roles of N6-methyladenosine (m^6^A) methylation in RNA processing and non-communicable diseases. Cancer Gene Ther. 2024;31(10):1439–1453. 38839892 10.1038/s41417-024-00789-1

[12] Viegas IJ, de Macedo JP, Serra L, De Niz M, Temporão A, Silva Pereira S, Mirza AH, Bergstrom E, Rodrigues JA, Aresta-Branco F, et al. N^6^-methyladenosine in poly(A) tails stabilize VSG transcripts. Nature. 2022;604(7905):362–370. 35355019 10.1038/s41586-022-04544-0 PMC9150445

[13] Meng S, Liu H, Xu J, Deng C, Qian X, Chu S, Zhu WG, Zhu J, Yong H, Li Z, et al. PRMT5-mediated ALKBH5 methylation promotes colorectal cancer immune evasion via increasing CD276 expression. Research. 2025;8:0549. 39781264 10.34133/research.0549 PMC11707101

[14] Yan Y, Luo A, Liu S, Cai M, Liu X, Zhang X, Zhang S, Liu Y, Zeng J, Xu X, et al. METTL3-mediated LINC00475 alternative splicing promotes glioma progression by inducing mitochondrial fission. Research. 2024;7:0324. 38405130 10.34133/research.0324 PMC10886067

[15] Baumgarten S, Bryant JM, Sinha A, Reyser T, Preiser PR, Dedon PC, Scherf A. Transcriptome-wide dynamics of extensive m^6^A mRNA methylation during *plasmodium falciparum* blood-stage development. Nat Microbiol. 2019;4(12):2246–2259. 31384004 10.1038/s41564-019-0521-7 PMC7611496

[16] Govindaraju G, Kadumuri RV, Sethumadhavan DV, Jabeena CA, Chavali S, Rajavelu A. N^6^-Adenosine methylation on mRNA is recognized by YTH2 domain protein of human malaria parasite *Plasmodium falciparum*. Epigenetics Chromatin. 2020;13(1):33. 32867812 10.1186/s13072-020-00355-7 PMC7457798

[17] Govindaraju G, Chavali S, Rajavelu A. *Plasmodium falciparum* YTH2 domain binds to m6A-containing mRNA and regulates translation. MBio. 2021;12(6): Article e0136721. 10.1128/mBio.01367-21 PMC8609348 34809465

[18] Holmes MJ, Padgett LR, Bastos MS, Sullivan WJ Jr. m6A RNA methylation facilitates pre-mRNA 3′-end formation and is essential for viability of *Toxoplasma gondii*. PLoS Pathog. 2021;17(7): Article e1009335. 34324585 10.1371/journal.ppat.1009335 PMC8354455

[19] Dagan Y, Yesharim Y, Bonneau AR, Frankovits T, Schwartz S, Reddien PW, Wurtzel O. m6A is required for resolving progenitor identity during planarian stem cell differentiation. EMBO J. 2022;41(21): Article e109895. 35971838 10.15252/embj.2021109895 PMC9627665

[20] Yesharim Y, Shwarzbard O, Barboy-Smoliarenko J, Cherian PV, Shachar R, Palavalli A, Vu HT, Schwartz S, Wurtzel O. Single-nucleotide m^6^A mapping uncovers redundant YTHDF function in planarian progenitor fate selection. EMBO J. 2026;45(3):749–788. 41484363 10.1038/s44318-025-00662-3 PMC12864844

[21] Narayan P, Rottman FM. An in vitro system for accurate methylation of internal adenosine residues in messenger RNA. Science. 1988;242(4882):1159–1162. 3187541 10.1126/science.3187541

[22] Wang Y, Li Y, Toth JI, Petroski MD, Zhang Z, Zhao JC. N^6^-methyladenosine modification destabilizes developmental regulators in embryonic stem cells. Nat Cell Biol. 2014;16(2):191–198. 24394384 10.1038/ncb2902 PMC4640932

[23] Liu J, Yue Y, Han D, Wang X, Fu Y, Zhang L, Jia G, Yu M, Lu Z, Deng X, et al. A METTL3-METTL14 complex mediates mammalian nuclear RNA N^6^-adenosine methylation. Nat Chem Biol. 2014;10(2):93–95. 24316715 10.1038/nchembio.1432 PMC3911877

[24] Ping X-L, Sun BF, Wang LU, Xiao W, Yang X, Wang WJ, Adhikari S, Shi Y, Lv Y, Chen YS, et al. Mammalian WTAP is a regulatory subunit of the RNA N6-methyladenosine methyltransferase. Cell Res. 2014;24(2):177–189. 24407421 10.1038/cr.2014.3 PMC3915904

[25] Schwartz S, Mumbach MR, Jovanovic M, Wang T, Maciag K, Bushkin GG, Mertins P, Ter-Ovanesyan D, Habib N, Cacchiarelli D, et al. Perturbation of m6A writers reveals two distinct classes of mRNA methylation at internal and 5′ sites. Cell Rep. 2014;8(1):284–296. 24981863 10.1016/j.celrep.2014.05.048 PMC4142486

[26] Lei K, Lin S, Yuan Q. N6-methyladenosine (m6A) modification of ribosomal RNAs (rRNAs): Critical roles in mRNA translation and diseases. Genes Dis. 2023;10(1):126–134. 37013049 10.1016/j.gendis.2021.10.005 PMC10066336

[27] Batista PJ, Molinie B, Wang J, Qu K, Zhang J, Li L, Bouley DM, Lujan E, Haddad B, Daneshvar K, et al. m^6^A RNA modification controls cell fate transition in mammalian embryonic stem cells. Cell Stem Cell. 2014;15(6):707–719. 25456834 10.1016/j.stem.2014.09.019 PMC4278749

[28] Luo GZ, MacQueen A, Zheng G, Duan H, Dore LC, Lu Z, Liu J, Chen K, Jia G, Bergelson J, et al. Unique features of the m^6^A methylome in *Arabidopsis thaliana*. Nat Commun. 2014;5(1):5630. 25430002 10.1038/ncomms6630 PMC4248235

[29] Schwartz S, Agarwala SD, Mumbach MR, Jovanovic M, Mertins P, Shishkin A, Tabach Y, Mikkelsen TS, Satija R, Ruvkun G, et al. High-resolution mapping reveals a conserved, widespread, dynamic mRNA methylation program in yeast meiosis. Cell. 2013;155(6):1409–1421. 24269006 10.1016/j.cell.2013.10.047 PMC3956118

[30] Linder B, Grozhik AV, Olarerin-George AO, Meydan C, Mason CE, Jaffrey SR. Single-nucleotide-resolution mapping of m6A and m6Am throughout the transcriptome. Nat Methods. 2015;12(8):767–772. 26121403 10.1038/nmeth.3453 PMC4487409

[31] Yesharim Y, Shwarzbard O, Barboy-Smoliarenko J, Shachar R, Palavalli A, Vu HT-K, Schwartz S, Wurtzel O. Single-nucleotide m^6^A mapping uncovers redundant YTHDF function in planarian progenitor fate selection. bioRxiv. 2025. 10.1101/2025.03.03.641144 PMC12864844 41484363

[32] Wang P, Doxtader KA, Nam Y. Structural basis for cooperative function of Mettl3 and Mettl14 methyltransferases. Mol Cell. 2016;63(2):306–317. 27373337 10.1016/j.molcel.2016.05.041 PMC4958592

[33] Qiu C, Hong Y, Cao Y, Wang F, Fu Z, Shi Y, Wei M, Liu S, Lin J. Molecular cloning and characterization of glutamine synthetase, a tegumental protein from *Schistosoma japonicum*. Parasitol Res. 2012;111(6):2367–2376. 23011789 10.1007/s00436-012-3092-6

[34] Wang J, Yu Y, Shen H, Qing T, Zheng Y, Li Q, Mo X, Wang S, Li N, Chai R, et al. Dynamic transcriptomes identify biogenic amines and insect-like hormonal regulation for mediating reproduction in *Schistosoma japonicum*. Nat Commun. 2017;8(1):14693. 28287085 10.1038/ncomms14693 PMC5355954

[35] Chen R, Wang J, Gradinaru I, Vu HS, Geboers S, Naidoo J, Ready JM, Williams NS, DeBerardinis RJ, Ross EM, et al. A male-derived nonribosomal peptide pheromone controls female schistosome development. Cell. 2022;185(9):1506–1520.e17. 35385687 10.1016/j.cell.2022.03.017 PMC9058237

[36] Gobert GN, Moertel L, Brindley PJ, McManus DP. Developmental gene expression profiles of the human pathogen *Schistosoma japonicum*. BMC Genomics. 2009;10(1):128. 19320991 10.1186/1471-2164-10-128 PMC2670322

[37] Ramirez AS, Kowal J, Locher KP. Cryo-electron microscopy structures of human oligosaccharyltransferase complexes OST-A and OST-B. Science. 2019;366(6471):1372–1375. 31831667 10.1126/science.aaz3505

[38] Anderson L, Amaral MS, Beckedorff F, Silva LF, Dazzani B, Oliveira KC, Almeida GT, Gomes MR, Pires DS, Setubal JC, et al. *Schistosoma mansoni* egg, adult male and female comparative gene expression analysis and identification of novel genes by RNA-Seq. PLoS Negl Trop Dis. 2015;9(12): Article e0004334. 26719891 10.1371/journal.pntd.0004334 PMC4699917

[39] Moertel L, McManus DP, Piva TJ, Young L, McInnes RL, Gobert GN. Oligonucleotide microarray analysis of strain- and gender-associated gene expression in the human blood fluke, Schistosoma japonicum. Mol Cell Probes. 2006;20(5):280–289. 16647836 10.1016/j.mcp.2006.02.002

[40] LoVerde PT, Niles EG, Osman A, Wu W. Schistosoma mansoni male–female interactions. Can J Zool. 2004;82(2):357–374.

[41] Moffat D, Kusel JR. Fluorescent lipid uptake and transport in adult *Schistosoma mansoni*. Parasitology. 1992;105(Pt 1):81–89. 1437279 10.1017/s0031182000073716

[42] Rogers SH, Bueding E. Anatomical localization of glucose uptake by *Schistosoma mansoni* adults. Int J Parasitol. 1975;5(3):369–371. 1126790 10.1016/0020-7519(75)90086-7

[43] Skelly PJ, Wilson RA. Making sense of the schistosome surface. Adv Parasitol. 2006;63:185–284. 17134654 10.1016/S0065-308X(06)63003-0

[44] Miller CL, Day TA, Bennett JL, Pax RA. *Schistosoma mansoni*: L-glutamate-induced contractions in isolated muscle fibers; evidence for a glutamate transporter. Exp Parasitol. 1996;84(3):410–419. 8948330 10.1006/expr.1996.0129

[45] Lence T, Akhtar J, Bayer M, Schmid K, Spindler L, Ho CH, Kreim N, Andrade-Navarro MA, Poeck B, Helm M, et al. m^6^A modulates neuronal functions and sex determination in *Drosophila*. Nature. 2016;540(7632):242–247. 27919077 10.1038/nature20568

[46] Finkel D, Groner Y. Methylations of adenosine residues (m^6^A) in pre-mRNA are important for formation of late simian virus 40 mRNAs. Virology. 1983;131(2):409–425. 6318439 10.1016/0042-6822(83)90508-1

[47] Huang G, Zhang F, Xie D, Ma Y, Wang P, Cao G, Chen L, Lin S, Zhao Z, Cai Z. High-throughput profiling of RNA modifications by ultra-performance liquid chromatography coupled to complementary mass spectrometry: Methods, quality control, and applications. Talanta. 2023;263: Article 124697. 37262985 10.1016/j.talanta.2023.124697

[48] Dominissini D, Moshitch-Moshkovitz S, Salmon-Divon M, Amariglio N, Rechavi G. Transcriptome-wide mapping of N^6^-methyladenosine by m^6^A-seq based on immunocapturing and massively parallel sequencing. Nat Protoc. 2013;8(1):176–189. 23288318 10.1038/nprot.2012.148

[49] Martin M. Cutadapt removes adapter sequences from high-throughput sequencing reads. EMBnet J. 2011;17(1):10–12.

[50] Luo F, Yin M, Mo X, Sun C, Wu Q, Zhu B, Xiang M, Wang J, Wang Y, Li J, et al. An improved genome assembly of the fluke *Schistosoma japonicum*. PLoS Negl Trop Dis. 2019;13(8): Article e0007612. 31390359 10.1371/journal.pntd.0007612 PMC6685614

[51] Dobin A, Davis CA, Schlesinger F, Drenkow J, Zaleski C, Jha S, Batut P, Chaisson M, Gingeras TR. STAR: ultrafast universal RNA-seq aligner. Bioinformatics. 2013;29(1):15–21. 23104886 10.1093/bioinformatics/bts635 PMC3530905

[52] Wei Z, exomePeak2: Peak calling and differential analysis for MeRIP-Seq, R package version 1.12.0, Bioconductor (2023); https://bioconductor.org/packages/exomePeak2/

[53] Cui X, Wei Z, Zhang L, Liu H, Sun L, Zhang SW, Huang Y, Meng J. Guitar: an R/bioconductor package for gene annotation guided transcriptomic analysis of RNA-related genomic features. BioMed Res Int. 2016;2016(1): Article 8367534. 27239475 10.1155/2016/8367534 PMC4864564

[54] Micallef L, Rodgers P. eulerAPE: Drawing area-proportional 3-Venn diagrams using ellipses. PLoS One. 2014;9(7): Article e101717. 25032825 10.1371/journal.pone.0101717 PMC4102485

[55] Cantalapiedra CP, Hernandez-Plaza A, Letunic I, Bork P, Huerta-Cepas J. eggNOG-mapper v2: Functional annotation, orthology assignments, and domain prediction at the metagenomic scale. Mol Biol Evol. 2021;38(12):5825–5829. 34597405 10.1093/molbev/msab293 PMC8662613

[56] Ou J, Wolfe SA, Brodsky MH, Zhu LJ. motifStack for the analysis of transcription factor binding site evolution. Nat Methods. 2018;15(1):8–9. 29298290 10.1038/nmeth.4555

[57] Machanick P, Bailey TL. MEME-ChIP: Motif analysis of large DNA datasets. Bioinformatics. 2011;27(12):1696–1697. 21486936 10.1093/bioinformatics/btr189 PMC3106185

[58] Livak KJ, Schmittgen TD. Analysis of relative gene expression data using real-time quantitative PCR and the 2^−ΔΔCT^ method. Methods. 2001;25(4):402–408. 11846609 10.1006/meth.2001.1262

[59] Altschul SF, Gish W, Miller W, Myers EW, Lipman DJ. Basic local alignment search tool. J Mol Biol. 1990;215(3):403–410. 2231712 10.1016/S0022-2836(05)80360-2

[60] Tamura K, Stecher G, Kumar S. MEGA11: Molecular Evolutionary Genetics Analysis version 11. Mol Biol Evol. 2021;38(7):3022–3027. 33892491 10.1093/molbev/msab120 PMC8233496

[61] Nicholas KB, Nicholas HB, Deerfield DWI, Nicholas K, Nicholas H, Deerfield D, Nicholas KB, Nicholas KR, Deerfield D, Deerfield DW, et al. GeneDoc: Analysis and visualization of genetic variation. EMBnet News. 1997;4:1–4.

[62] Subramanian B, Gao S, Lercher MJ, Hu S, Chen WH. Evolview v3: A webserver for visualization, annotation, and management of phylogenetic trees. Nucleic Acids Res. 2019;47(W1):W270–W275. 31114888 10.1093/nar/gkz357 PMC6602473

[63] Cogswell AA, Collins JJ 3rd, Newmark PA, Williams DL. Whole mount in situ hybridization methodology for *Schistosoma mansoni*. Mol Biochem Parasitol. 2011;178(1–2):46–50. 21397637 10.1016/j.molbiopara.2011.03.001 PMC3102561

[64] Wang X, Fang C, Cheng G. Genome-wide identification and functional characterization of GPCR family genes reveal their key roles in the vitellarium development and egg production in *Schistosoma japonicum*. Parasit Vectors. 2025;18(1):286. 40676690 10.1186/s13071-025-06929-2 PMC12273374

[65] Liu J, Zhu L, Wang J, Qiu L, Chen Y, Davis RE, Cheng G. *Schistosoma japonicum* extracellular vesicle miRNA cargo regulates host macrophage functions facilitating parasitism. PLoS Pathog. 2019;15(6): Article e1007817. 31163079 10.1371/journal.ppat.1007817 PMC6548406

[66] Zhang WN, Zhang P, Liu M, Ren CP, Jia XM, Huang DK, Gui L, Shen JJ. Worm morphology of *Schistosoma japonicum* using confocal laser scanning microscopy. J Helminthol. 2012;86(3):317–322. 21810283 10.1017/S0022149X11000447

[67] Zhang J, Shen W, Liu F, He H, Han S, Luo L. Fracture-healing effects of Rhizoma Musae ethanolic extract: An integrated study using UHPLC-Q-Exactive-MS/MS, network pharmacology, and molecular docking. PLoS One. 2025;20(1): Article e0313743. 39808649 10.1371/journal.pone.0313743 PMC11731732

[68] Cheng G, Fu Z, Lin J, Shi Y, Zhou Y, Jin Y, Cai Y. In vitro and in vivo evaluation of small interference RNA-mediated gynaecophoral canal protein silencing in *Schistosoma japonicum*. J Gene Med. 2009;11(5):412–421. 19288459 10.1002/jgm.1314 PMC7166781

[69] Shan T, Lu Z, Xia T, Wang X, Liu L, Fang C, Li S, Zheng Y, Cheng G. Non-parasite genome encoded virus-like RNAs reprogram the pathogenicity of human blood flukes. Nat Commun. 2025;17(1): Article 2995. 41453891 10.1038/s41467-025-67822-1 PMC13036067

[70] Wu T, Hu E, Xu S, Chen M, Guo P, Dai Z, Feng T, Zhou L, Tang W, Zhan LI, et al. clusterProfiler 4.0: A universal enrichment tool for interpreting omics data. Innovation. 2021;2(3): Article 100141. 34557778 10.1016/j.xinn.2021.100141 PMC8454663

[71] Zhang J, Li H, Tao W, Zhou J. GseaVis: An R package for enhanced visualization of gene set enrichment analysis in biomedicine. Med Res. 2025;1(1):131–135.

[72] Shannon P, Markiel A, Ozier O, Baliga NS, Wang JT, Ramage D, Amin N, Schwikowski B, Ideker T. Cytoscape: A software environment for integrated models of biomolecular interaction networks. Genome Res. 2003;13(11):2498–2504. 14597658 10.1101/gr.1239303 PMC403769

[73] Chin C-H, Chen S-H, Wu H-H, Ho C-W, Ko M-T, Lin C-Y. cytoHubba: Identifying hub objects and sub-networks from complex interactome. BMC Syst Biol. 2014;8(Suppl 4):S11. 25521941 10.1186/1752-0509-8-S4-S11 PMC4290687

