# Supplementary material for: N6-Methyladenosine on Key Messenger RNAs Governs Reproductive Development and Metabolic Adaptation in Human Blood Fluke
Source: Research (Wash D C). 2026 Jul 1;9:1328. doi: 10.34133/research.1328 (PMC13319631; doi:10.34133/research.1328)
Supplement: Supplementary 1 — Figs. S1 to S9 Tables S1 to S5 Videos S1 and S2 [file research.1328.f1.zip › Supplementary_Figs information-20260616.docx]

Supplementary Materials for

**N^6^-methyladenosine on Key mRNAs Governs Reproductive Development and Metabolic Adaptation in Human Blood Fluke**

Bikash Ranjan Giri *et al.*

*Corresponding author.

Guofeng Cheng, Shanghai Tenth People’s Hospital, Institute for Infectious Diseases and Vaccine Development, Tongji University School of Medicine, Shanghai, China; School of Life Sciences and Technology, Tongji University, Shanghai, China; Clinical Center for Brain and Spinal Cord Research, Tongji University, Shanghai, China; Affiliated Shanghai Blue Cross Brain hospital, School of Medicine, Tongji University, Shanghai 200020, China.

Cizhong Jiang, Key Laboratory of Spine and Spinal Cord Injury Repair and Regeneration of the Ministry of Education, Orthopaedic Department of Tongji Hospital, School of Life Sciences and Technology, Tongji University, Shanghai, China

Email: [chengguofeng@tongji.edu.cn](mailto:chengguofeng@tongji.edu.cn)

[czjiang@tongji.edu.cn](mailto:czjiang@tongji.edu.cn)

**This PDF file includes:**

**Figs. S1-S9**

**Tables S1-S5**

**Movies S1-S2**

**
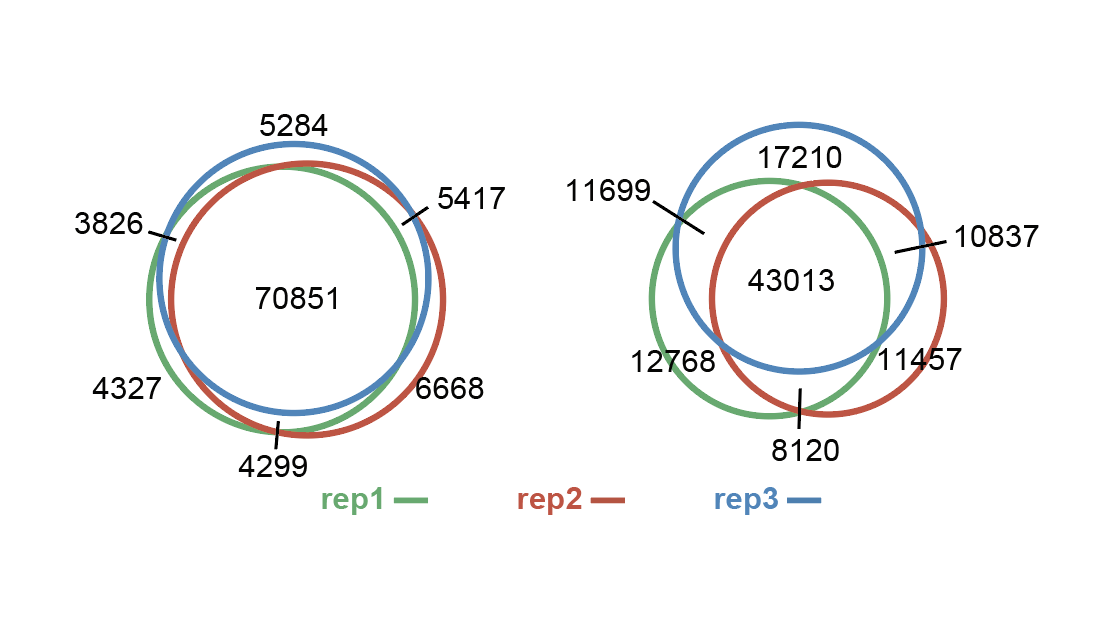
**

Male

Female

**Fig. S1.** Overlapping of the identified m^6^A peaks between males and females among three biological replicates.

**A**

**
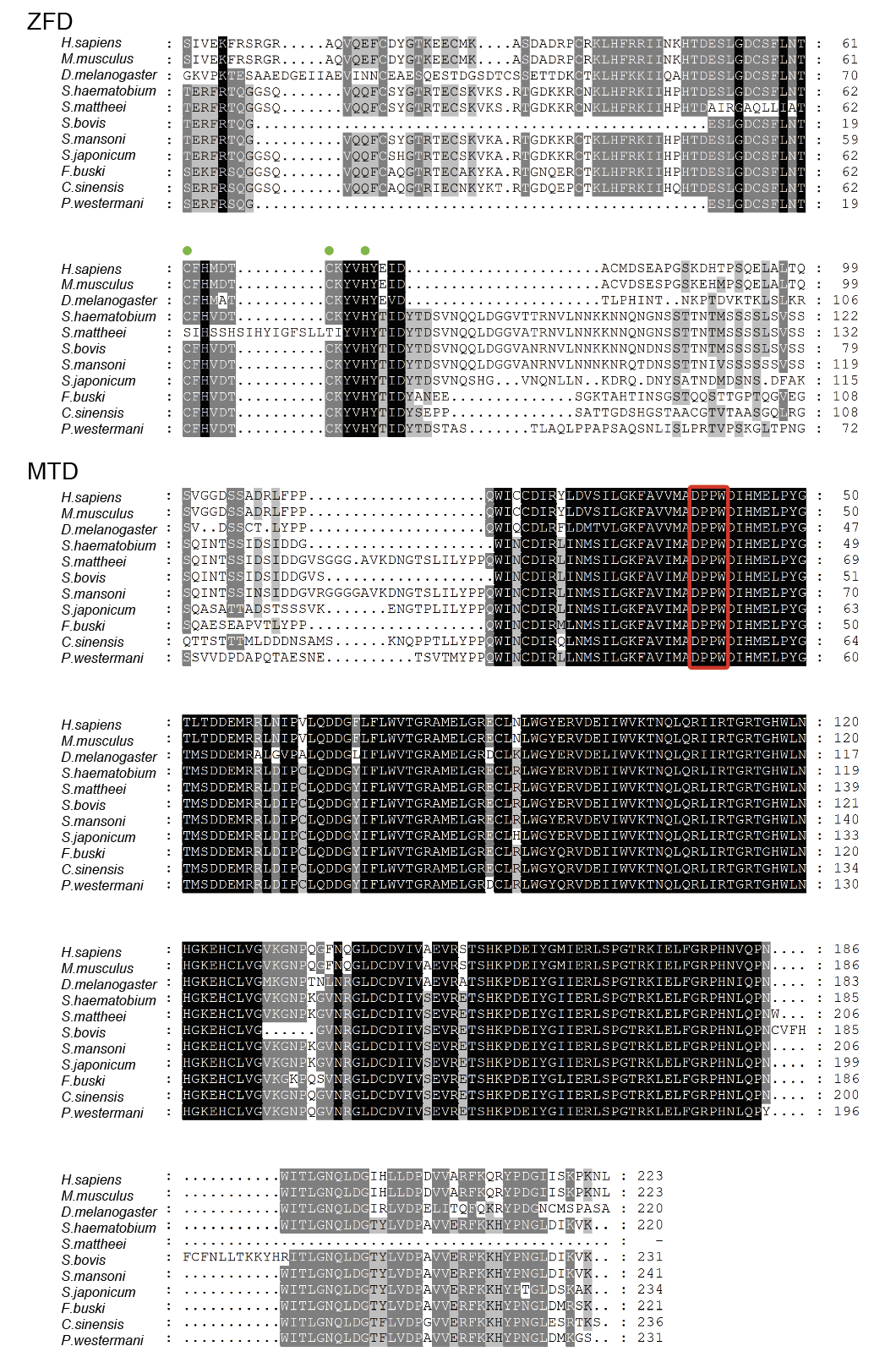
**

**B**

**
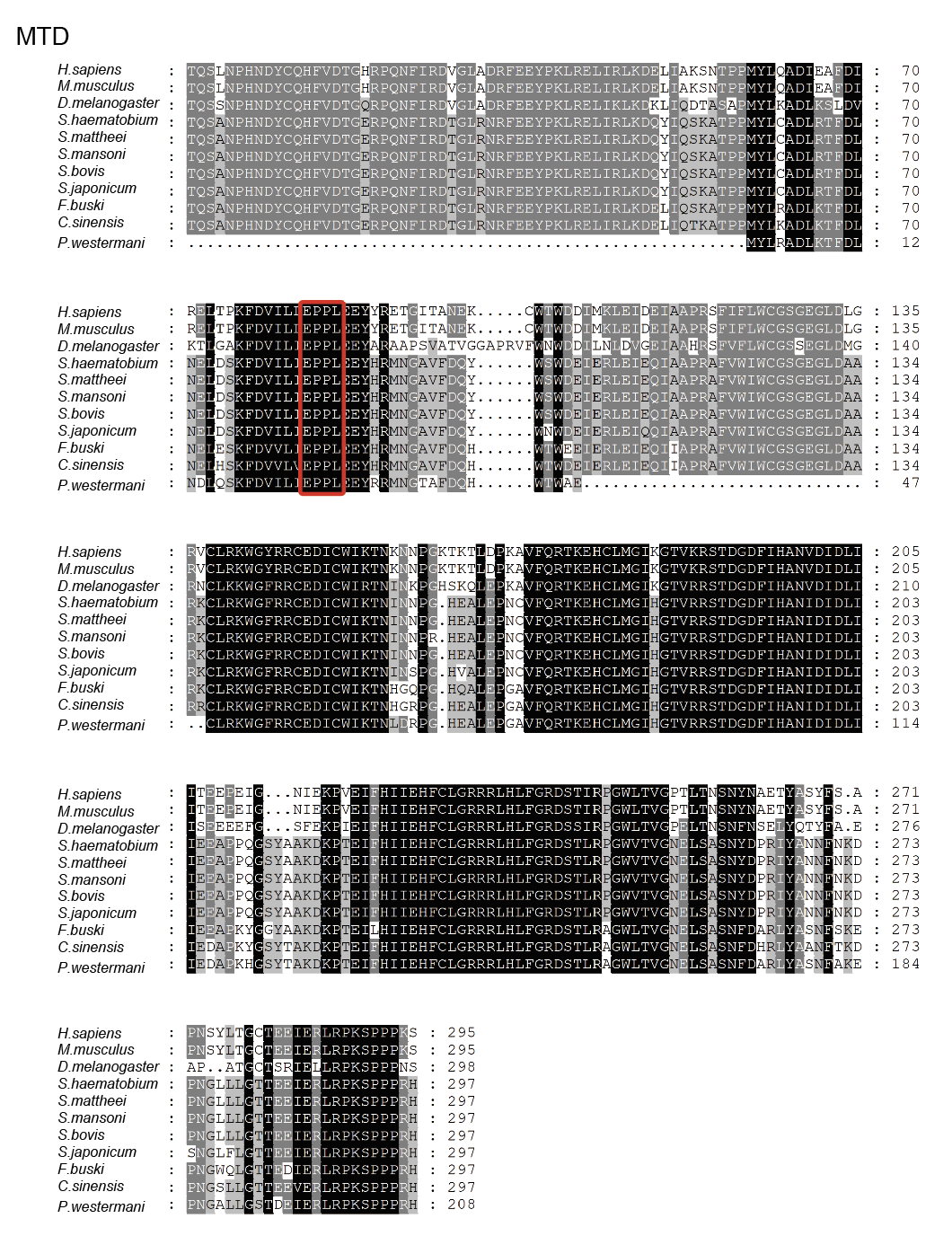
**

**Fig. S2.** Analysis of Conserved domains of m^6^A writers in *S. japonicum.*

(A) Conservation of *S. japonicum* METTL3 across different species. The ZFD domain of METTL3 contained two CCCH-type zinc finger motifs (ZnF1 and ZnF2). Green dots represent highly conserved zinc-coordinating residues. The MTD domain also showed high conservation, and the DPPW motif is boxed in red. (B) Conservation of *S. japonicum* METTL14 across different species. MTD region is highly conserved and the EPPL motif is boxed in red.

**
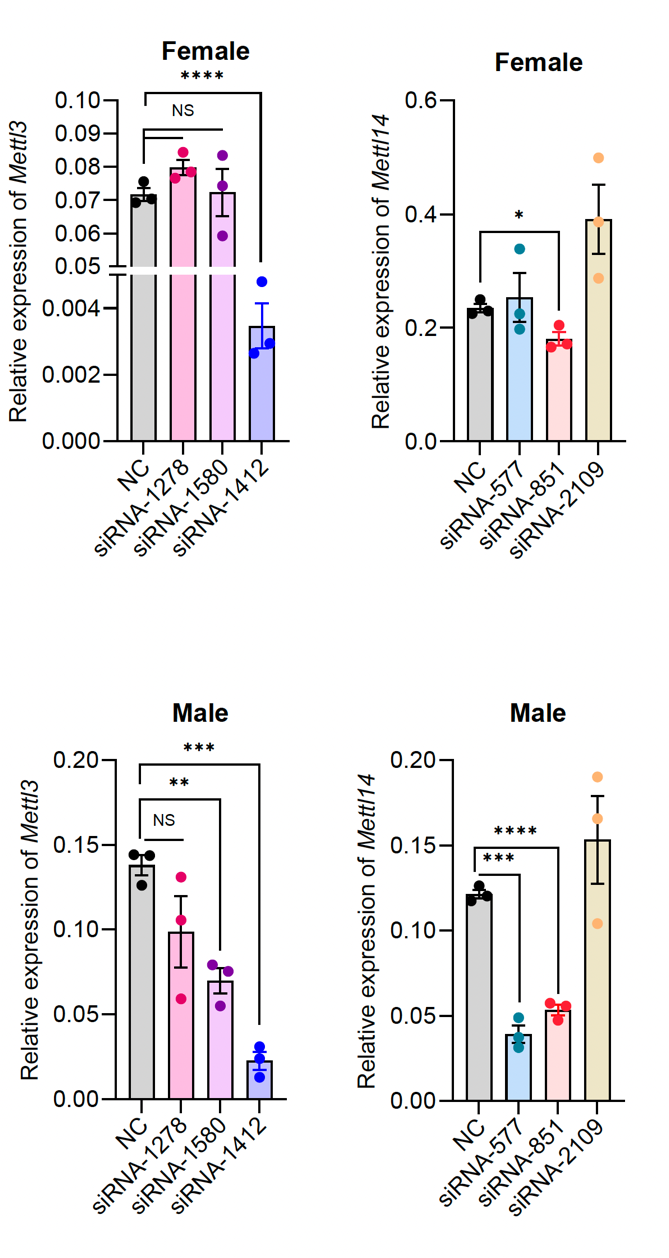
**

**Fig. S3.** Assessment of the best siRNAs for silencing *Mettl3*/*Mettl14* in adult worms. Data illustrate representative results indicating mean and standard deviation obtained from an experiment conducted in triplicate. Significant differences between the NC (Negative control siRNA) and siRNA-treated groups were analyzed by Student’s t-test (**P*< 0.05; ***P* < 0.01; ****P* < 0.001; *****P* < 0.0001).


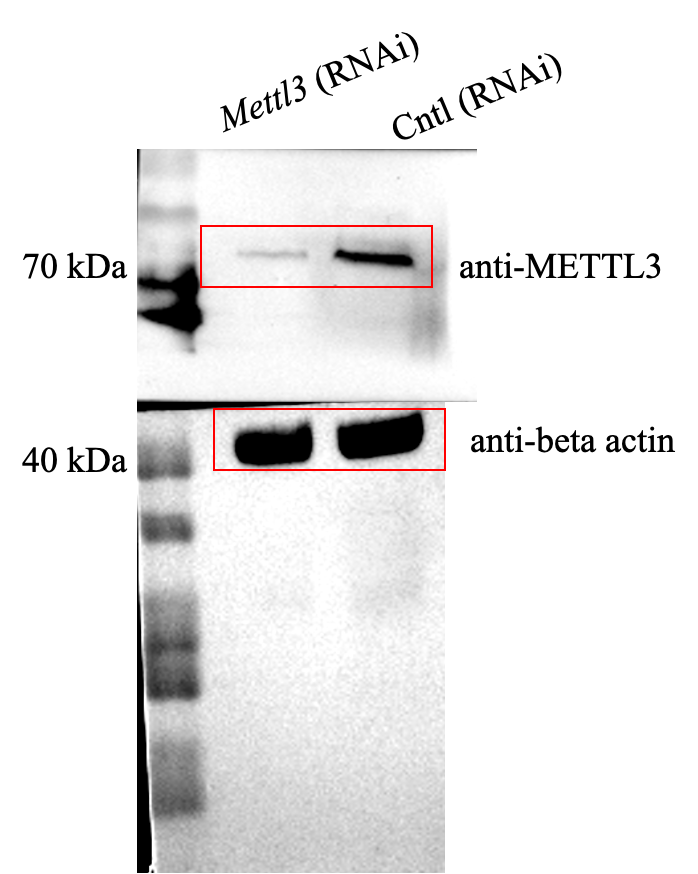


**Fig. S4.** Western blot analysis of decreased *Mettl3* at protein levels.


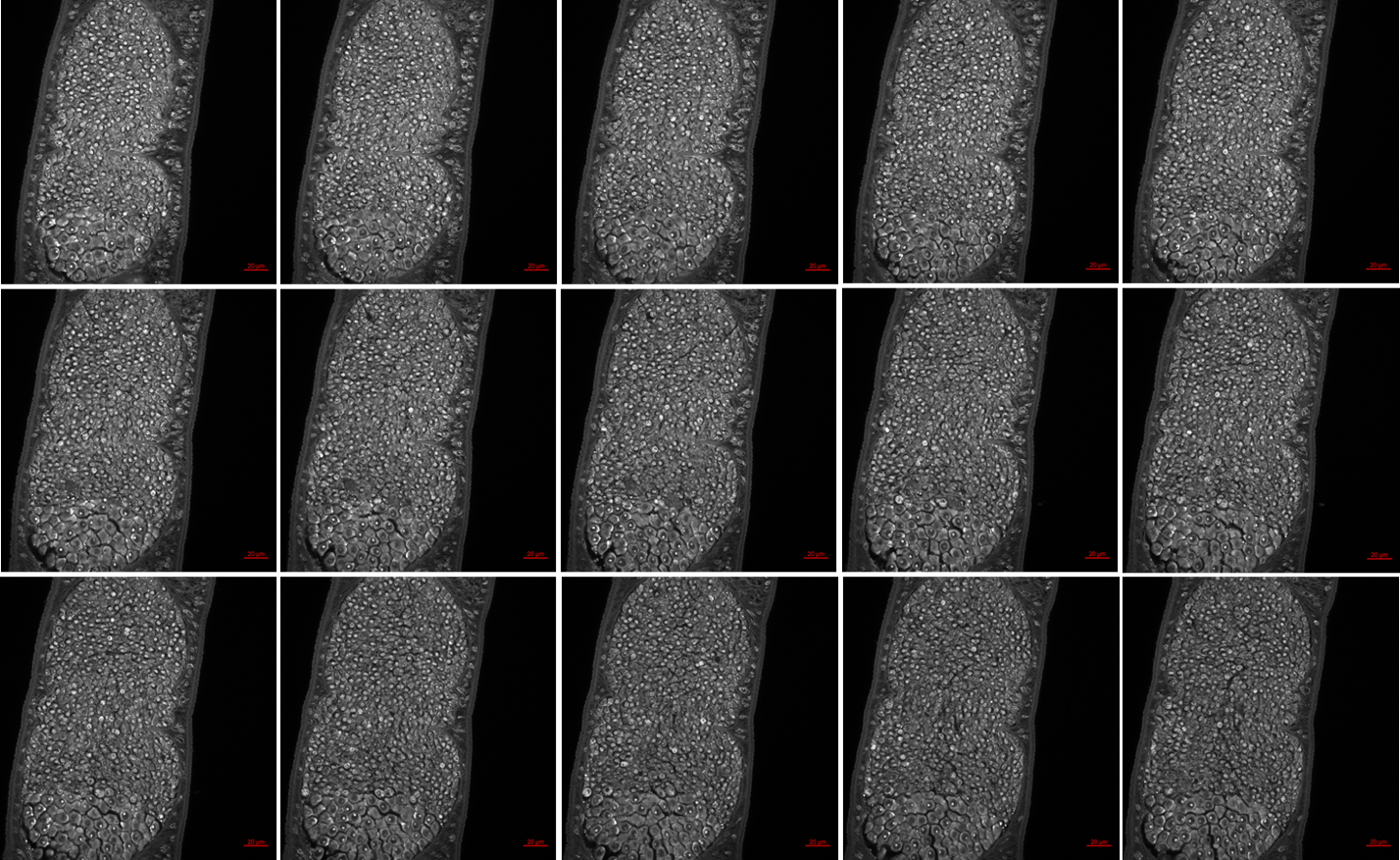
**A**

**
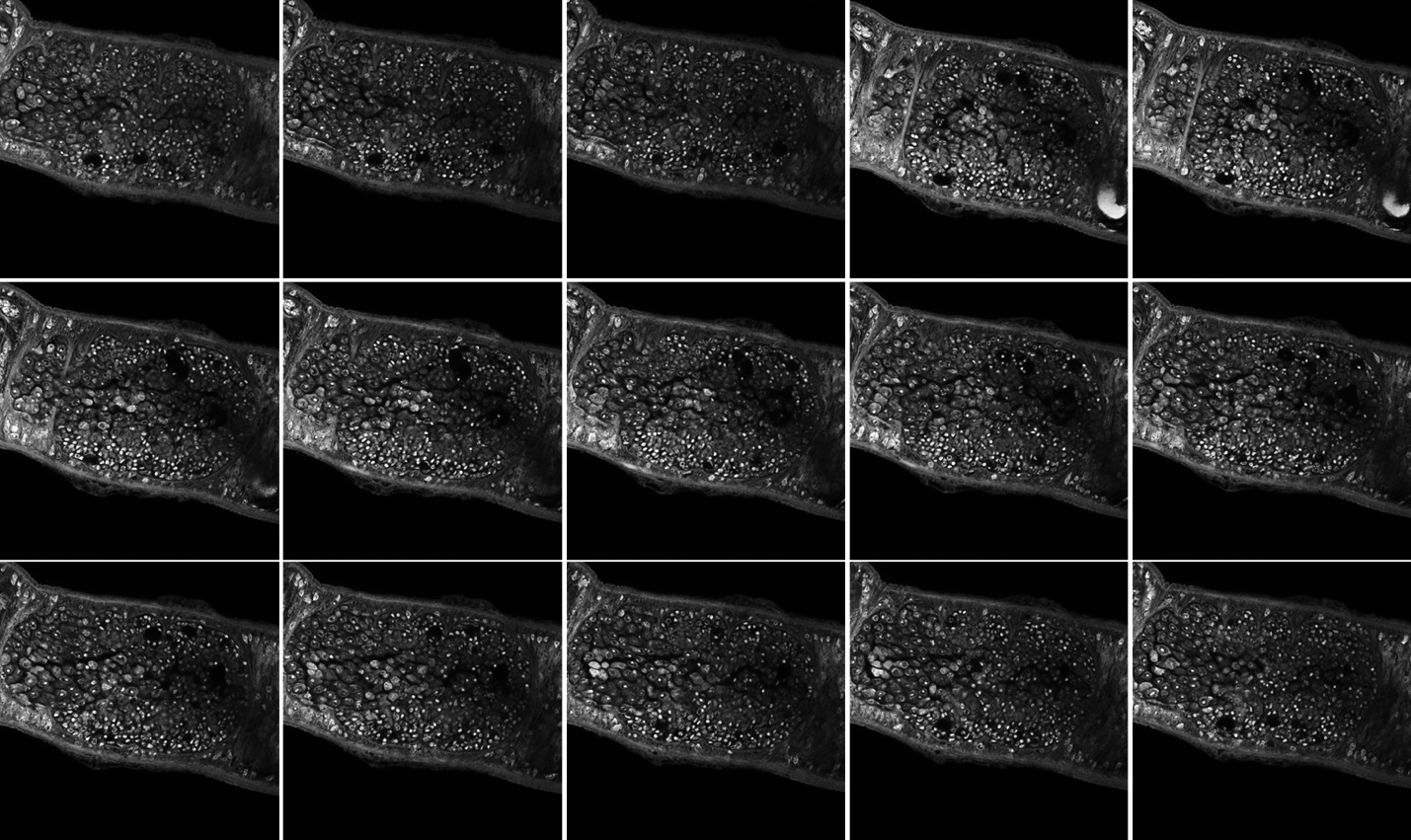
B**

**
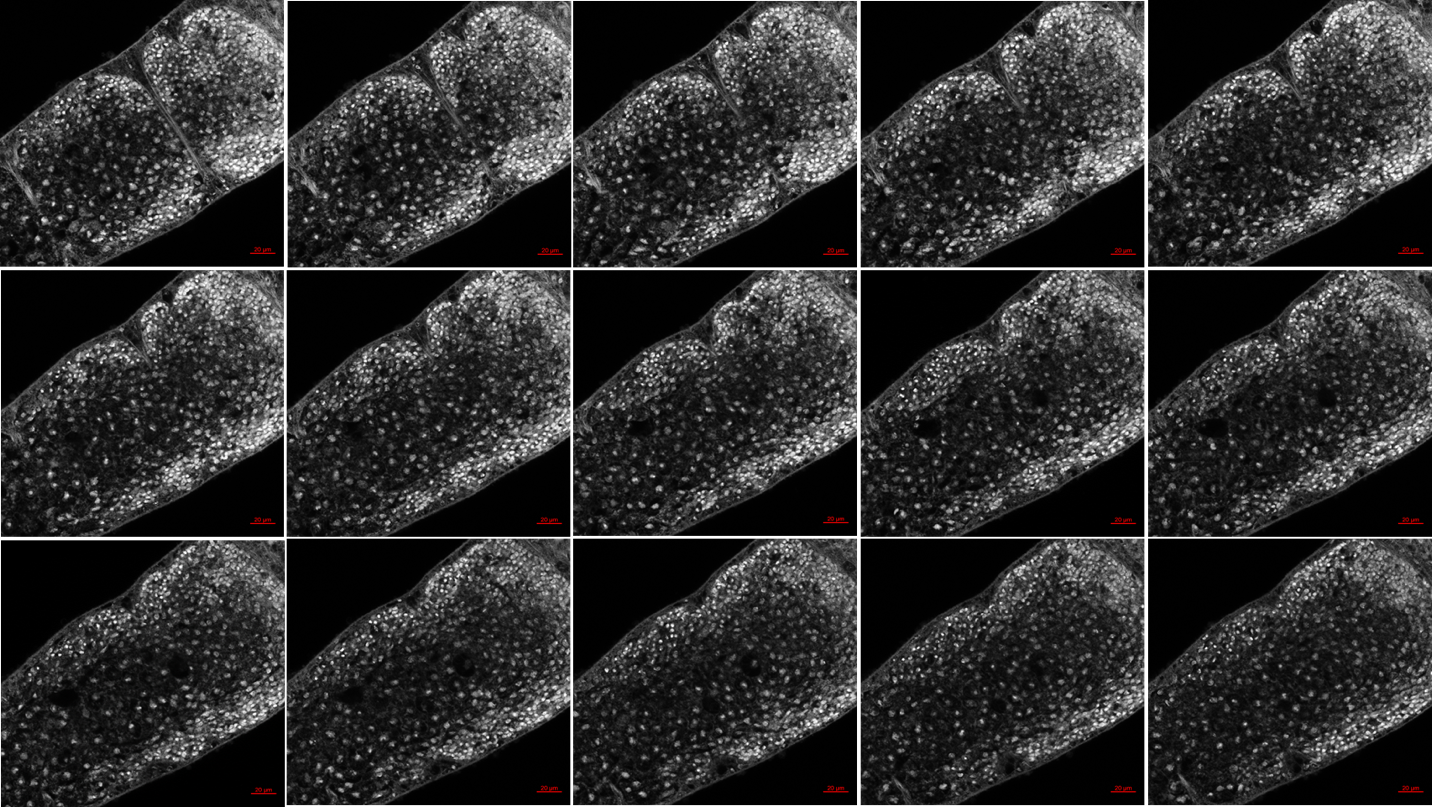
C**

**Fig. S5.** Z-stack of *S*. *japonicum* ovaries treated with control siRNA (A), *Mettl3* siRNA (B), and *Mettl14* siRNA (C).

**
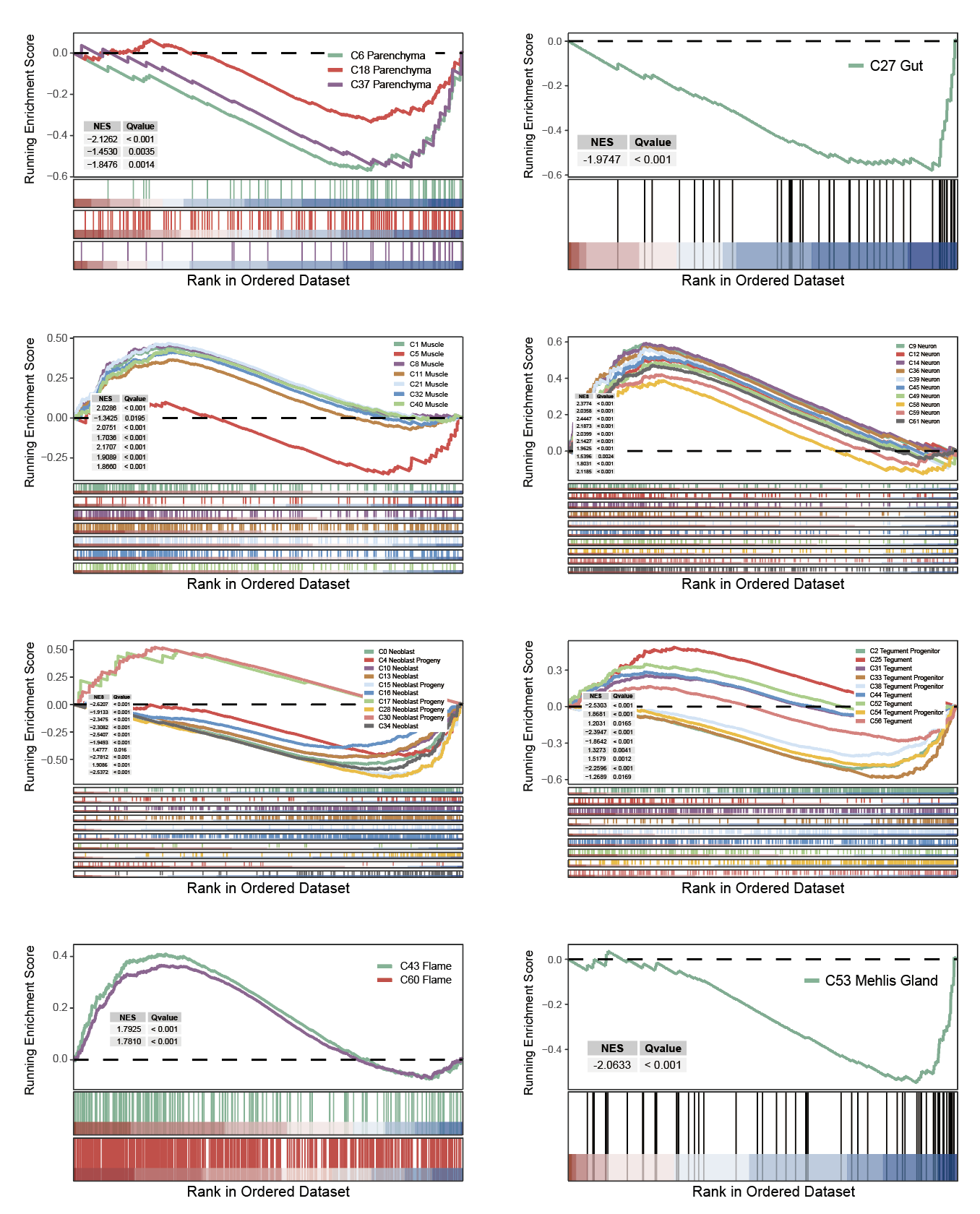
**

**Fig. S6.** GSEA results for the genes with reduced m^6^A levels in *Mettl3* KD females.

**
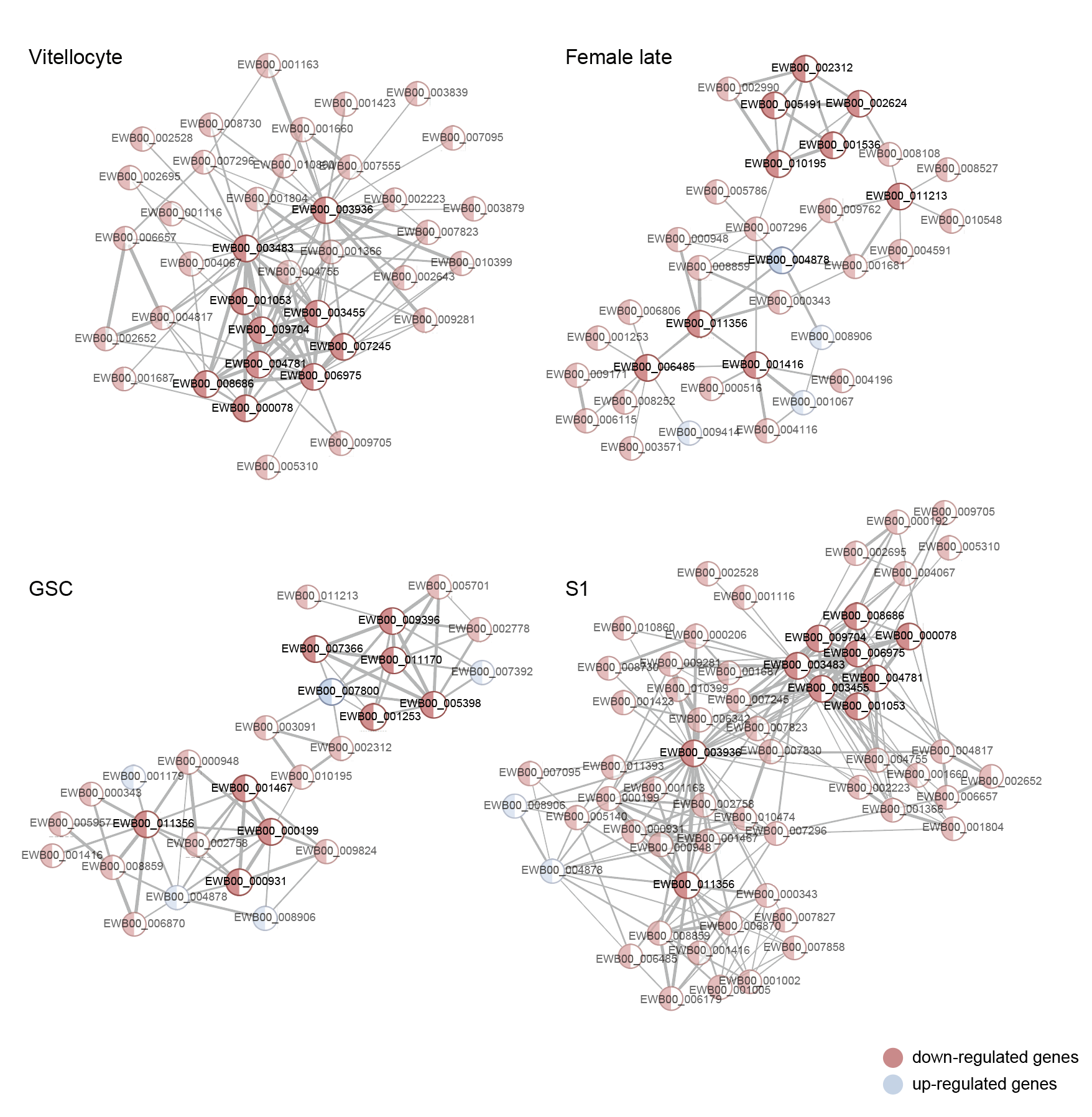
**

**Fig. S7.** Protein-protein interaction map (PPI) networks of the genes exhibiting reduced m⁶A levels following *Mettl3* KD in females.


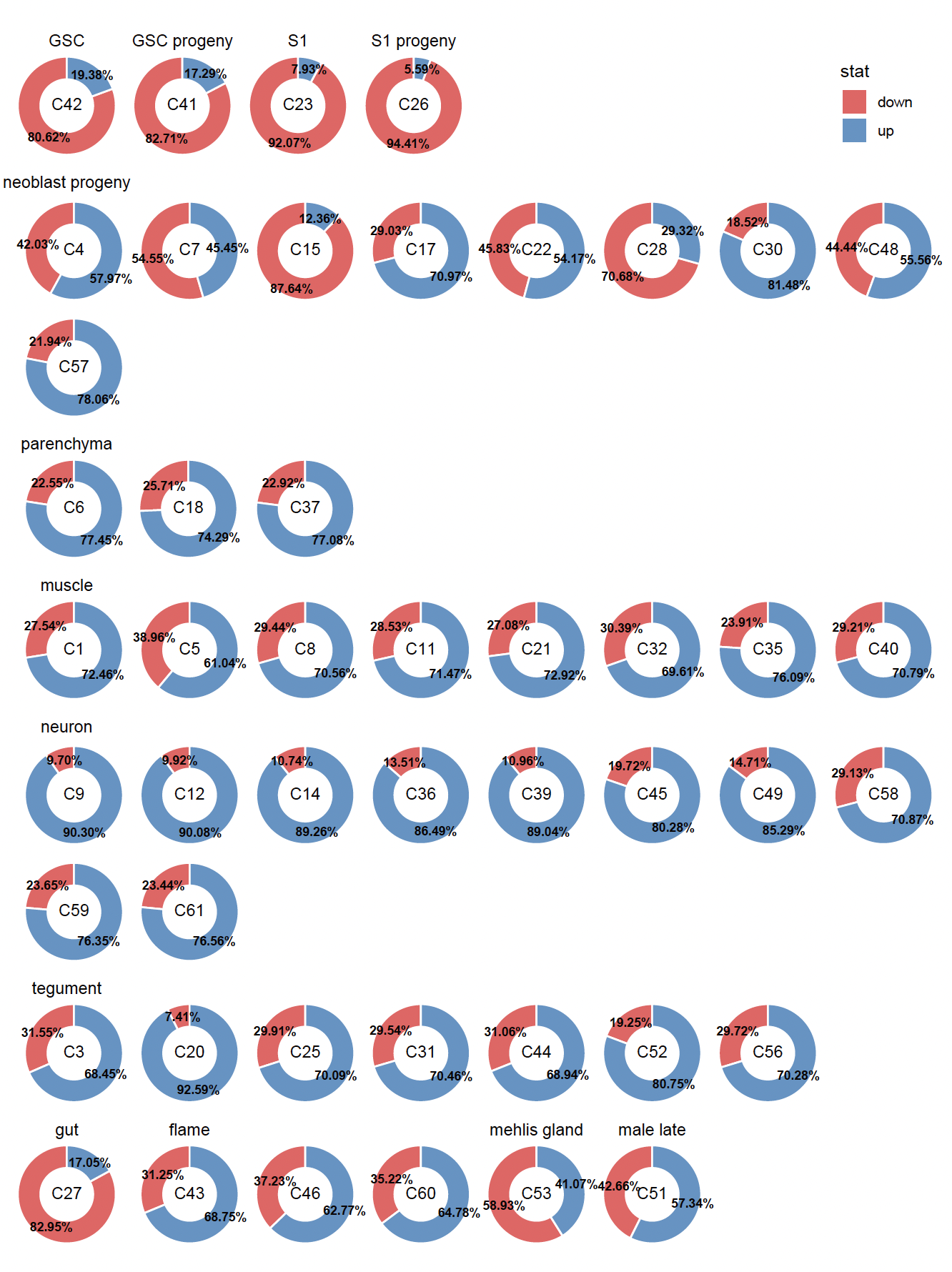


**Fig. S8.** Integration with our scRNA-seq data showing downregulated genes enriched in neoblasts, tegument progenitors, germline stem cells (GSCs), gut lineages and others.

**A**


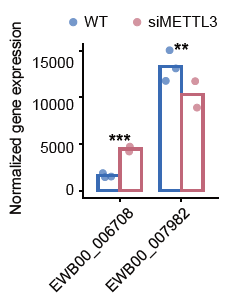


**B**
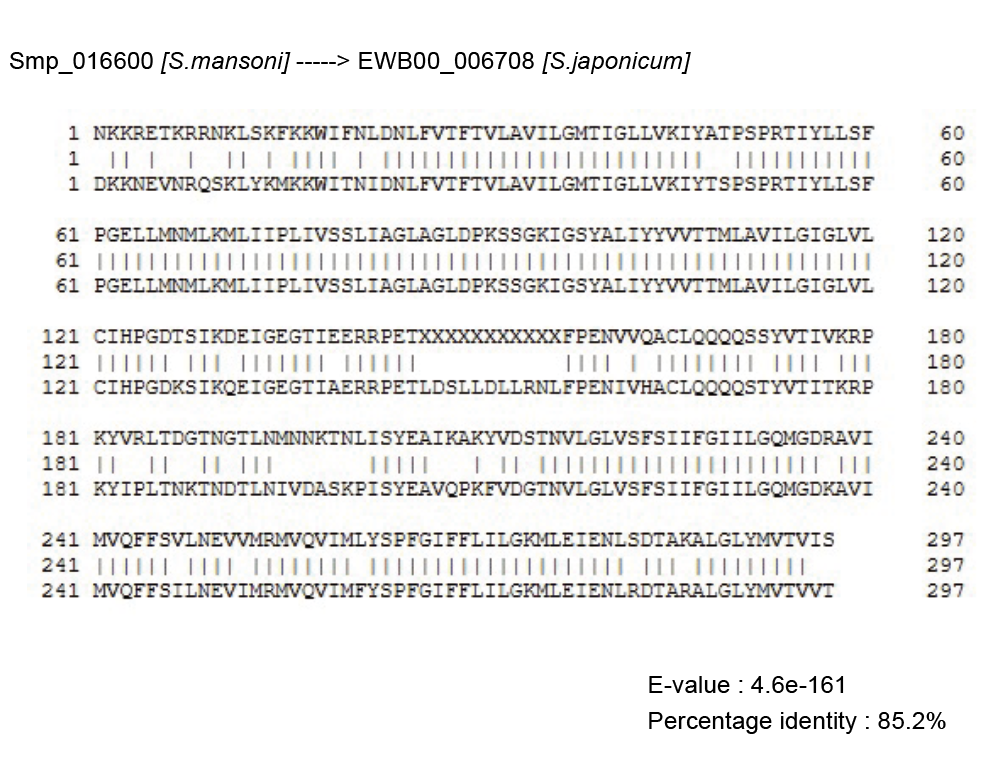


**Fig. S9.** Analysis of the expressions of two glutamine related genes in *Mettl3* suppressed males. (A) Expression levels of two metabolically relevant m^6^A target genes upon *Mettl3* suppressed males. EWB00_007982 encodes glutamine synthetase and EWB00_006708 encodes glutamate transporter. **q < 0.01; ***q < 0.001 (Wald test). (B) BLASTP analysis of the identity between *S. japonicum* glutamate transporter (EWB00_006708) and its *S. mansoni* ortholog (Smp_016600).

**Table S1**. The list of siRNA sequences and primers used in the present study.

**Table S2**. The list of identified m^6^A peaks in females.

**Table S3**. The list of identified m^6^A peaks in males.

**Table S4.** The list of differentially expressed genes between the *Mettl3* KD males and the controls.

**Table S5**. The list of suppressed genes in Fig 6G

**Video S1.** Worm motility in *Mettl3* KD males.

**Video S2.** Worm motility in control siRNA treated males.
